# The Role of Functional Neuroimaging in Pre-Surgical Epilepsy Evaluation

**DOI:** 10.3389/fneur.2014.00031

**Published:** 2014-03-24

**Authors:** Francesca Pittau, Frédéric Grouiller, Laurent Spinelli, Margitta Seeck, Christoph M. Michel, Serge Vulliemoz

**Affiliations:** ^1^Presurgical Epilepsy Evaluation Unit, Neurology Department, University Hospital of Geneva, Geneva, Switzerland; ^2^Department of Radiology and Medical Informatics, University Hospital of Geneva, Geneva, Switzerland; ^3^Functional Brain Mapping Laboratory, Department of Fundamental Neurosciences, University of Geneva, Geneva, Switzerland

**Keywords:** focal epilepsy, EEG-fMRI, ESI, PET, SPECT, MRS, functional neuroimaging

## Abstract

The prevalence of epilepsy is about 1% and one-third of cases do not respond to medical treatment. In an eligible subset of patients with drug-resistant epilepsy, surgical resection of the epileptogenic zone is the only treatment that can possibly cure the disease. Non-invasive techniques provide information for the localization of the epileptic focus in the majority of cases, whereas in others invasive procedures are required. In the last years, non-invasive neuroimaging techniques, such as simultaneous recording of functional magnetic resonance imaging and electroencephalogram (EEG-fMRI), positron emission tomography (PET), single photon emission computed tomography (SPECT), electric and magnetic source imaging (MSI, ESI), spectroscopy (MRS), have proved their usefulness in defining the epileptic focus. The combination of these functional techniques can yield complementary information and their concordance is crucial for guiding clinical decision, namely the planning of invasive EEG recordings or respective surgery. The aim of this review is to present these non-invasive neuroimaging techniques, their potential combination, and their role in the pre-surgical evaluation of patients with pharmaco-resistant epilepsy.

## Introduction

Epilepsy is one of the most frequent chronic neurological disorders, with an incidence of 50/100,000/year and a prevalence of 0.5–1% (1, 2) in the Western society. One-third of patients with epilepsy are resistant to anti-epileptic drug treatment (3, 4) and this outcome is already evident after the first 12 months (5). The majority of patients with epilepsy suffer from focal seizures caused by an abnormal neuro-electrical activity of a focal epileptogenic zone that can subsequently spread to other brain regions. This concept is intimately linked to the correlation between early ictal signs and symptoms, electro-physiological activity, and structural lesion [anatomo-electro-clinical correlation, (6)]. Epilepsy surgery is the only treatment that can possibly cure epilepsy in patients with pharmaco-resistant epilepsy; this option should therefore be considered as soon as pharmacoresistance is manifest or even before in clear cases. In well-selected patients, epilepsy surgery is highly effective: the best outcome is obtained for temporal lobe surgery (up to 84% seizure freedom), followed by lesional extra-temporal epilepsy (up to 74%). The persistence of good outcome (over 50% seizure freedom) has been reported at a longer follow-up (5 or 10 years) (7, 8). Nevertheless, postponing surgery is a major problem and mostly reflects concerns of the medical community that diverge from the available evidence and expert guidelines (9, 10). Misconception of eligibility criteria, variable perception of pharmacoresistance and potential outcome of drug treatment, fear of complications, and/or presumably witnessed complications are the most common causes (11–13).

The risk and benefit assessment prior to epilepsy surgery needs to consider the morbidity and mortality associated with chronic pharmaco-resistant epilepsy. A systematic review on studies between 1990 and 2008 reported a mortality of 0.4% in patients with temporal and 1.2% with extra-temporal lobe resection and major neurological complications due to epilepsy surgery in 4.7% of patients (mostly as major visual field defect) (14). On the other hand, several studies have shown that patients with active epilepsy have mortality three times higher than the general population (expected age- and sex-adjusted mortality) (15, 16). Pre-surgical assessment should therefore be offered to any patient with persistence of seizures despite two treatment trials in sufficiently high dosages (17). The aim of epilepsy surgery is to remove the epileptogenic zone with the preservation of the eloquent areas (18). If the focus cannot be indentified or if it is impossible to remove it because it is located in eloquent cortex, then pre-surgical work-up will not lead to resective surgery; nevertheless in most cases, pre-surgical work-up allows a diagnosis with an inherent prognosis, and the evaluation of other treatment options. In the last 10–20 years, non-invasive functional neuroimaging techniques have proved their usefulness in the pre-surgical assessment of epilepsy, especially thanks to their continuous development. Non-invasive techniques provide information for the identification of the epileptic focus in the majority of cases; nevertheless the use of intracranial EEG is additionally required in 25–50% of patients (19–21). The core of pre-surgical evaluation consists of accurate clinical evaluation, interictal and ictal EEG, dedicated structural MRI with an epilepsy protocol, and neuropsychological assessment.

The aim of this review is to present different functional imaging techniques, currently used to localize the epileptic focus in the pre-surgical evaluation of patients with drug-resistant focal epilepsy. This review will focus on the functional imaging techniques and will not consider advanced structural techniques such as post-processing of structural MRI, quantitative analysis, and diffusion imaging.

## Methods

An electronic literature search was conducted for articles on this topic regarding human subjects (in all age groups). Sources searched included PubMed and relevant books. To summarize the literature search strategy: (1) words used in the searches included the text words and subject headings of “seizure*, epilep*, localizing value, spectroscopy (or MRS), positron emission tomography (or PET), single photon emission computed tomography (or SPECT), simultaneous functional MRI (or fMRI), and EEG (or EEG-fMRI), electric and magnetic source imaging (or MSI, ESI).” The words were searched independently and in combination. (2) PubMed was also checked for articles already retrieved through other searches. For each citation considered, the abstract was read (when available), and articles were excluded if they were outside the scope of the review. Studies published only in abstract form, letters, and technical reports were excluded. Also excluded were any articles reporting the use of the explored techniques for other indications. The bibliography of each of the retrieved papers was examined to identify relevant references that could have been missed by electronic search. The findings were described taking into account the limit of words and the critical insight of the authors.

## Electric Source Imaging

### Principle

Electroencephalography (EEG) has long been the key diagnostic tool for epileptologists and remains at the heart of the pre-surgical evaluation. In the past decades, EEG analysis has been revolutionized by digital and computer technology. Beyond multichannel temporal oscillations EEG data can be represented as time-series of scalp potential maps that vary across time with the temporal resolution in the order of milliseconds (22). Electric source imaging (ESI) allows the estimation of the electric sources underlying these maps (23–25). Several studies have now confirmed its role as an accurate tool for estimating the source of focal epilepsy (26–29).

Electric source imaging is obtained by (i) building a head-model to describe the propagation of electro-magnetic fields through the head and (ii) solving the inverse problem of source localization, which consists of inferring the location of the generators of brain activity from signals detected outside the head. The methodological details and a review of head models and inverse solutions are beyond the scope of this clinically oriented paper. Most important with respect to the head-model in the context of epilepsy is to use the patient’s own brain anatomy in order to account for cerebral abnormalities (30–32). Concerning the inverse solution, equivalent dipole or distributed solutions have been applied to epilepsy. While equivalent dipole models assume that the momentary brain electrical activity is confined to a few focal regions, distributed inverse solutions estimate the current density distribution in the whole brain, usually restricted to the gray matter of the individual brain (33, 34). Multiple algorithms for distributed inverse solutions have been developed, each integrating specific *a priori* mathematical and biophysical assumptions (35). These have been the subject of several recent reviews (24, 28, 36).

An important factor to consider is how precisely the electric field is sampled at the head surface. The localization of interictal spikes is significantly improved by increasing the number of electrodes from a standard 31-electrode montage to 128 electrodes (37). Sampling the electric field below the top of the ears is also fundamental to localize generators in the inferior and medial parts of temporal lobes (38). The recent introduction of EEG caps with more than 200 electrodes (up to 256) and easy ways of application has made high-density EEG available in the clinical neurophysiology laboratory (39). A recent reappraisal of the skull conductivity toward higher values reduces the skull “blurring” effect and suggests that even a higher density of electrodes could be beneficial to sampling the brain activity on the scalp. There is evidently a stronger case in children where the conductivity is higher than in adults.

### Interictal localization

The majority of studies of ESI (and MSI, see next section) in epilepsy have focused on localizing interictal spikes rather than seizures. Indeed interictal spikes are usually more frequent than seizures, can be averaged together in order to improve the signal-to-noise ratio, and their spatio-temporal dynamics are simpler (40). The temporal resolution of EEG allows differentiating the generation of a spike from its propagation: concerning ESI, the EEG map at its 50% rising phase is selected for source localization, as the IED peak is contaminated by propagation (34). ESI of interictal spikes attempts to localize the irritative zone. The clinical usefulness of these techniques depends both on its absolute accuracy and on the value of the irritative zone as a surrogate for the seizure-onset zone and the epileptogenic zone (which needs to be removed for obtaining seizure freedom (18)). When other clinical informations are integrated in the analysis, the localization of spikes appears to be a valid index of the seizure-onset zone and the epileptogenic zone (41).

Several clinical studies have shown the reliability of ESI in a wide patient spectrum, adults, and children with non-lesional epilepsy or large lesions (32, 36, 38, 42, 43). In a recent ESI study of non-lesional extra-temporal epilepsy (44) only focal interictal, but not ictal discharges, were highly associated with excellent surgical outcome, indicating that the careful analysis and localization of interictal spikes lead to important information for the surgical result. Another recent study validated by intracranial recordings in 33 patients has shown that ESI of interictal spikes is an excellent surrogate for localizing the seizure-onset zone, which is a key to the surgical planning (45), thereby confirming previous intracranial EEG studies of spikes and seizure-onset localization (41). Like the results of each diagnostic tool, the results of ESI must always be integrated within the patient’s overall clinical, neurophysiological, and radiological picture, in order to assess its reliability in estimating the epileptogenic zone and identify electroclinical discrepancies that might explain discordant ESI localizations.

The accuracy of ESI has been assessed in large groups of patients with different epilepsy types using intracranial EEG as a gold standard. Concordance between dipolar sources and intracranial EEG has been found in a high percentage of cases with either temporal or frontal lobe epilepsy (46, 47). ESI is able to localize correctly mesial temporal discharges (i.e., 4–5 cm deep sources) in most of the patients. This has been shown by recording interictal discharges simultaneously from scalp and foramen ovale electrode recordings of the mesial temporal structures (48, 49) as well as in cognitive tasks involving the hippocampus studied with scalp and invasive EEG (50, 51). Recently, simultaneous high-density scalp EEG and intracranial EEG reports have supported these findings (52).

The accuracy of ESI can be also assessed by comparing the results with the resected brain volume as a function of post-operative outcome: the validation is proven if the localization of the studied technique falls within the resection and if the patient is subsequently seizure-free. This approach makes sense in the clinical context of epilepsy surgery, although other factors can play a role in this evaluation (extension of the resection, neuro-surgeons choice). Regarding ESI, in the largest study investigating 152 subsequently operated patients (39), a sensitivity of 84% and specificity of 88% were found, superior to those of more classical localization techniques, such as the presence of a lesion on structural MRI (76% sensitivity, 53% specificity) or focal abnormalities on nuclear functional imaging (interictal PET 69% sensitivity, 44% specificity, ictal SPECT 58% sensitivity, 47% specificity). False positive cases on MRI or PET could be caused by multifocal abnormalities and non-lesional MRI offers obvious false negative cases. Of note, only patients with interictal spikes detected on high resolution EEG were included in the high resolution ESI group, while low resolution ESI was obtained for the others. Importantly, ESI performed as well for patients with temporal lobe epilepsy as it did for patients with extra-temporal lobe epilepsy. The inferior and mesial temporal brain foci were correctly localized when individual head models are used and by providing a sufficient electrode number. Regarding this last point, the accuracy of ESI decreased when ESI was performed based on the standard, 32-channel EEG recordings instead of the 128- or 256-channel high-density EEG systems (sensitivity and specificity around 60%). Other studies with smaller patient numbers support these findings (27, 53). Low density ESI is a valuable additional imaging tool, which only requires a good quality EEG with a well-thought electrode distribution, a standard 3D anatomical MRI from clinical epilepsy imaging protocol, and processing with freeware tools. Therefore, ESI could be performed with little additional cost in any epilepsy surgery center.

### Ictal localization

Also ictal activity can be localized by ESI (54–56). This type of analysis showed good results when computed in the time domain (26, 57) or by using the dominant frequency at the seizure-onset (58). The best concordance between ESI and Stereo-EEG (SEEG) has been obtained for ictal spike patterns and for paroxysmal fast activities on the scalp (55). Different findings, even with lower spatial sampling, have been validated with intracranial EEG recordings (59–61). It is important to apply ESI at the very beginning of the seizure because of fast propagation of the activity and the increasing contamination by muscular artifacts. High-density EEG caps that allow recording for at least 24 h, including period of sleep, will likely increase the number of seizures to be analyzed with ESI. Currently, ictal ESI remains difficult to perform given the low signal-to-noise ratio fueled by artifacts, non-stationary patterns, and the paucity of the recorded events. Post-processing methods, attempting at extracting ictal patterns from the EEG have shown some promises but require further validation (62).

## Magneto-Encephalography and Magnetic Source Imaging

### Principle

Magneto-encephalography (MEG) differs from EEG by the fact that it detects magnetic instead of electric fields produced by neuronal currents, using sensors homogeneously placed around the head (63, 64). The amplitude of magnetic fields for physiological brain activity is very low (from femto-teslas to pico-teslas, eight orders of magnitude smaller than the magnetic field of the earth). For this reason very sensitive magnetometers (superconducting quantum interference devices) and strict shielding from outside interferences are required.

Both MEG and EEG measure cerebral activity in real time, but they measure different physical properties of this activity. This leads to differences in their sensitivity to different configurations of neural generators, but they do not provide independent information about the neuronal generators in the brain as initially postulated [for in-depth discussions, see Ref. (65, 66)]. Magnetic fields diffuse across skull and scalp with no appreciable distortion, whereas electrical potentials are distorted due to the different electrical conductivities due to variations of skull thickness, cranial foramina, previous craniotomies, etc. (63). This allows recording MEG in patients with traumatic or post-operative skull breeches without the major limitations encountered with EEG/ESI, which would require very accurate modeling of the skull anatomy. However, MEG is only sensitive to the activity of neurons located tangentially to the skull. Therefore, MEG reflects the activity in cortical sulci or in the major brain fissures (sylvian, interhemispheric) that is unbalanced by the contralateral surface. On the contrary, EEG is able to record the activity of neurons regardless of their orientation (67), although it can substantially affect the spatial distribution of the observed EEG. An additional difference between the two techniques is that MEG sensors are attached to the machine and not to the patient’s head, making MEG very sensitive to patient motion. For this reason, MEG recordings cannot last more than a couple of hours, and it is difficult to perform studies of seizures or in sleep, and in young children or non-cooperative patients.

### Interictal studies

Magnetic source imaging can influence the strategy for implanting intracranial electrodes (68, 69). In these two studies, the implantation strategy was decided twice for each patient: first after reviewing the results of all investigations except MSI, and then again after showing the results of MSI. MSI brought to change the implantation strategy in 23–33% of cases, showing that this technique supplies non-redundant information to a significant proportion of patients who have already undergone multiple non-invasive testing modalities. A recent study on 21 MRI-negative patients has demonstrated, by using a recently described method that allows a delineation of the brain spiking volume [volumetric imaging of epileptic spikes, VIES, (70)], that patients having focal VIES-MEG results are good surgical candidates and the implantation strategy should include VIES results. In contrast, patients with non-focal MEG results have less probably a localized seizure-onset zone; in these cases SEEG is not advised unless clear localizing information is provided by other pre-surgical investigation methods (71). The concept that MSI should be taken into account when defining the strategy for resective surgery is supported by other previous findings of a positive association between inclusion of MSI results in the resection and further seizure freedom (72, 73).

Agirre-Arrizubieta et al. (74) reported concordant localization findings with MSI in 90% of lateral temporal spikes, 80% of interhemispheric and peri-central spikes, and 60% of superior frontal spikes. However, concordant localization was only 40% for orbito-frontal spikes, and 0% for medial temporal spikes. Similar results were obtained by another study by Knowlton et al. (75) who found the concordance between MSI and the seizure-onset zone to be about 80% in patients with lateral temporal lobe epilepsy and 45% in those with medial temporal lobe or extra-temporal lobe epilepsy. The poor performance with mesial temporal and basal frontal discharges can be explained by the difficulty of MEG to visualize deep-seated electrical sources (28). Also studies from the groups of Oishi (76) and Huiskamp (77) have shown, by comparing MEG spikes with Electro-CorticoGraphy spikes that MEG sensitivity varies for different regions in the brain. Knowlton et al. (75) have reported that the performance of MSI was on average similar to that of PET and ictal SPECT. When comparing MSI with depth electrode recordings (69, 78), MEG source localization did show excellent spatial accuracy, especially for neocortical sources.

### Ictal sources

Few cases of seizures occasionally captured during an MEG recording (79) have been also described. The impossibility to record for more than a few hours, limits the ability to record ictal events with MEG.

## ESI/MSI Comparison

The comparative clinical value of ESI and MSI remains unsettled and controversial. To date, no study has investigated simultaneously recorded interictal spike, with similar numbers of sensors and head coverage with an undisputed gold standard. Studies published until now comparing high-density MEG systems against (at most) moderate resolution EEG recordings artificially tip the balance in favor of MEG (80–82). MEG and EEG are sensitive to different physical features of neural activity, and the two techniques can therefore bring complementary information with different strengths and weaknesses (66). Given their complementary nature, there are many reasons to believe that EEG/MEG combination with high spatial sampling of both modalities could be valuable in specific difficult clinical situations.

## Simultaneous EEG-fMRI

### Principle

The first fMRI signal modifications related to ictal activity were obtained without concurrent EEG recording (83). By comparing images acquired during seizure and during interictal period, BOLD (Blood Oxygen Level Dependant) signal changes measured by fMRI were observed in regions nearby the epileptic focus. Other fMRI-only studies have confirmed that we can observe BOLD signal variation in concordance with the epileptic focus during simple partial seizures (84) and even in subclinical seizures (85). These results demonstrated the possibility to locate epileptic results in fMRI during ictal or interictal state. To improve the usefulness of this technique, simultaneous temporal detection of epileptic events was needed.

The combination of the temporal resolution of EEG and of the spatial resolution of fMRI offers the opportunity to locate non-invasively the epileptic focus and to better understand the epileptic networks (86, 87). Simultaneous EEG and fMRI recordings (EEG-fMRI) can detect cerebral hemodynamic changes related to interictal epileptiform discharges (IEDs) identified on scalp EEG [(88); for methods: (89)].

While the first EEG-fMRI recordings were spike-triggered acquisitions (90–92), the development of effective removal of MRI-gradient artifacts and pulse-related artifacts allows to record continuously and simultaneously EEG during fMRI (93), offering much improved modelization of the BOLD signal.

### Interictal imaging

Multiple studies have demonstrated BOLD signal changes mostly in areas tightly coupled with the region generating focal IEDs (94) and concordant with intracerebral findings (95). EEG-fMRI results have proved to be reproducible between scans and more sensitive at higher field scanners (96). The reliability of IEDs-related BOLD responses has been assessed (97) and the importance of an accurate marking and classification of IEDs has been demonstrated (98, 99).

Deep generators have been successfully identified in patients with gray matter heterotopias, illustrating the whole-brain coverage of the technique (100). In malformations of cortical development, EEG-fMRI may help to establish the role of the lesion in the epileptogenesis and to determine the potential surgical target (101). In nine patients with non-lesional frontal epilepsy, focus localization with EEG-fMRI has been subsequently confirmed by other imaging modalities or pathology (102).

A good post-surgical outcome has been linked to surgical removal including the BOLD changes (103). A recent study on 35 operated patients has shown that if the cortex concordant with the maximal BOLD changes was completely removed during surgery, the positive predictive value of seizure freedom (follow-up at 12 months minimum) was 70%, whereas if all the BOLD changes were outside the resection, the negative predictive value was 90.9% (104). In 11/23 patients with focal cortical dysplasia and BOLD responses to spikes, EEG-fMRI was able to provide help in predicting post-operative outcome: focal BOLD changes concordant with the intracranial EEG suggested a good prognosis (4/5 cases), in contrary to diffuse or multifocal BOLD changes (5/6 cases) (105). It has been shown that EEG-fMRI allows a more specific localization of the epileptic focus when compared with scalp EEG alone, in half of patients with epilepsy from heterogeneous etiology (106). In another study, 4/8 patients previously discounted from epilepsy surgery, EEG-fMRI was able to provide complementary information that changed the clinical management (107). All these studies, invasively validated in some patients, support EEG-fMRI as a clinically useful non-invasive tool in drug-resistant epilepsy to define the epileptic focus. However, the usefulness of the technique remains limited, mostly due to a lack of spikes during fMRI, calling for a continuing effort in methodological improvements. Clear-cut BOLD changes in the resection area appear rather specific for a good outcome while diffuse changes suggest extensive epileptic activity and a worse prognosis.

Other studies demonstrated that EEG-fMRI is an interesting tool to characterize the spatio-temporal dynamics of reflex epilepsies (108–110). Several studies on patients with different types of epilepsy using non-invasive or invasive techniques have shown that focal epilepsies are actually related to an abnormal function of a network of cortical and subcortical brain structures rather than to a single epileptogenic region (87, 111–115). The concept of epileptic network could be explained by the hypothesis that the areas activated or deactivated together with the irritative zone represent privileged areas of discharge propagation. An area that is currently considered as a common node in human IEDs is the anterior frontal part of the piriform cortex (110, 116, 117), called “area tempestas.” In animal kindling models of temporal lobe epilepsy, this is an epileptogenically sensitive area (118–120). These and many other findings open the way to the transition from the “focus era” to the “network era” and EEG-fMRI coupled with other non-invasive and invasive techniques offers interesting possibilities in this field. Indeed, epileptic activity can propagate very fast toward neighboring areas, but also toward more distant and even controlateral regions (117, 121, 122). The combination of EEG-fMRI with ESI could help to distinguish activation cluster related to initiation of IED from propagated areas (123–126). The estimated EEG source activity can be used to improve the model of IEDs-related BOLD response and may enhance the localization of the irritative zone (127).

To improve the temporal resolution of fMRI, a newly established fast fMRI sequence called magnetic-resonance-encephalography (MREG) has been recently published and provides a temporal resolution of around 100 ms (128), increasing the yield of EEG-fMRI in epilepsy. This study on 13 patients showed that the patients’ number in whom BOLD responses correlated with the spike topography was higher with MREG, compared to conventional echo planar imaging and that the *t*-values of the BOLD response in the spike field were also significantly higher with MREG. This tool might prove useful in the study of temporal relationships between the different regions of the network, although the temporal resolution will remain two orders of magnitude lower than EEG and MEG.

Studies with large cohorts revealed that no significant spike-related BOLD changes are observed in 40–70% of the EEG-fMRI recordings in patients with focal epilepsy (129, 130). The reasons are twofold: in many patients, the absence of spikes during fMRI acquisition precludes statistical analysis; while in other patients there may be no significant spike-related BOLD changes. One cause of absence of BOLD response could be an imperfect modeling. Concerning this issue, the inclusion of additional confounding variables can improve the modeling of MRI and is variably used across centers: large motion (131), cardiac activity (132), fluctuating physiological rhythms (133), sleep-specific activity (134), eye blinks, and swallowing (135).

**Figure 1:**
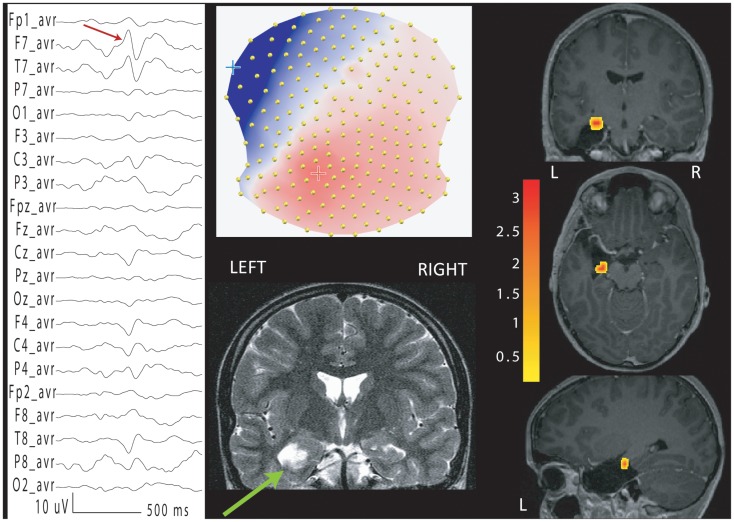
**EEG-fMRI in a patient with left temporal lobe epilepsy and left hippocampal cystic lesion (green arrow, bottom center)**. No IEDs were recorded during EEG-fMRI acquisition. IEDs acquired outside the scanner were averaged (the average spike is indicated by an arrow in average montage, on the left) and the corresponding voltage map was fitted (top center) to the EEG recorded inside the scanner [for method, see Ref. (139)]. The correlation coefficient was taken as a marker of epileptic activity and used as regressor for fMRI analysis. BOLD response showed a maximal activation in the left mesial temporal structures, that were subsequently surgically removed (co-registered EPI image with post-surgical MRI is shown on the right). The patient is seizure-free at 36 months follow-up.

Given the high proportion of patients without spikes or the absence of BOLD changes, alternative methods have been proposed. BOLD changes related to focal EEG slowing have been demonstrated (136). The detection of pathological EEG patterns using Independent Component Analysis of the EEG (137) increased the finding of concordant BOLD correlate of epileptic activity from 10 to 16/20 patients. In patients with concordant ICA components, BOLD and IEDs amplitude appear to be correlated (138). To overcome the problem of absence of IEDs in around 40% of the EEG-fMRI recordings, the use of patient-specific voltage map of epileptic spikes obtained from a long-term clinical EEG recording outside the MR scanner was proposed (Figure 1). The correlation of this epileptic topographic map with the EEG recorded inside the scanner was used to quantify the presence of ongoing epileptic activity and allow finding BOLD changes concordant with the presumed epileptic focus in 78% of the patients without IEDs during the simultaneous recordings, all with invasive validation (139). This method has subsequently been applied successfully in a pediatric group (140) offering the possibility to drastically improve the sensitivity of EEG-fMRI and to reduce the recording duration.

Different ways to analyze fMRI data, using various signal processing strategies such as independent component analysis or temporal clustering analysis, have been proposed to identify BOLD signatures of interictal activity independently from EEG (141–144). While such strategies represent promising efforts to address the problem of “non-spiking” EEG-fMRI, these methods still need to be validated in larger population of patients.

### Neuro-vascular coupling

Spike-related BOLD changes occur most often in the form of an increase, but a decrease can sometimes be observed, mostly (but not always) in regions remote to the epileptic focus (145). This phenomenon is still not fully understood (130, 146, 147). When located in the spike field, negative BOLD changes appear to have the same localization value as the positive BOLD (148). Localizing negative BOLD responses are rare, around 10% of all localizing BOLD responses. They are probably linked to a perturbation of the excitation/inhibition metabolic balance (149) and could be related to electro-physiological characteristics of the IEDs, like the presence of slow wave after the spike (150, 151). This could reflect a reduced neuronal activity following the spike, possibly the result of local inhibitory mechanism (152–154), or an impaired neuro-vascular coupling (151).

A modification in shape and/or in time of the canonical hemodynamic response function (HRF) has been reported in some patients especially in children (155–158). In some cases, hemodynamic changes even precede the IEDs (146, 147, 159–161). A study on a homogeneous cohort of children with benign epilepsy with centro-temporal spikes showed that the average BOLD response to centro-temporal spikes had significant differences to the canonical HRF, including an earlier onset and time-to-peak for the positive BOLD signal change, suggesting a possible role of the increase in synaptic activity preceding the spikes (154). Different other hypotheses have been proposed to explain this finding: (i) cerebral blood flow variations observed could be a source instead of a consequence of epileptic activity (162, 163); (ii) a neuronal discharge from deep structures not visible on scalp EEG occurs before the detected epileptic event (161); (iii) pre-spike metabolic responses could result from non-neuronal mechanisms including glia, and particularly astrocytes, which could be involved in epilepsy and in the regulation of cerebral blood flow (164–166). Different studies have demonstrated an inverted relationship between the resting state GABA measured by MR spectroscopy concentration, and amplitude of BOLD responses (167). New methods without assumptions on HRF based on the mutual information between EEG and fMRI (168) or on the decomposition of fMRI into independent components have been applied to focal epilepsy (169–171).

### Ictal imaging

Ictal EEG-fMRI is challenging due to the possible movements of the patients and to the low probability to record a seizure during the acquisition. Nevertheless, some studies demonstrated its usefulness for better understanding the hemodynamic correlates involved in the generation and the propagation of the seizures (162, 172–175). The simultaneous recording of video-EEG during ictal fMRI may help to detect BOLD changes associated to the different phases of the seizure (176). While ictal recordings remain serendipitous, longer scanner time and recruitment efforts have managed to build interesting case series with valuable insights into the seizure dynamics. The clinical value of such investigation remains difficult to assess except in patients with extremely frequent seizures.

### Simultaneous intracranial EEG and fMRI

Several studies also demonstrated the possibility to record simultaneously intracranial EEG and fMRI offering the opportunity to study epileptic networks with unprecedented sensitivity and specificity (177–180). Local and remote BOLD changes to very focal epileptic activity were demonstrated. Given the partial spatial sampling inherent to intracranial EEG, EEG-fMRI could offer a whole-brain investigation of epileptic networks in selected patients with intracranial electrodes. It is also a unique window to investigate neuro-vascular coupling of healthy and pathological implanted brain structures.

## Positron Emission Tomography

In the late seventies, PET has been the first functional technique applied for the localization of epileptic focus in patients with drug-resistant epilepsy (181). It typically uses radio-labeled fluoro-deoxy-glucose (FDG-PET) to show images of interictal brain glucose metabolism. Areas of functional deficit related to epileptic activity are characterized by reduced interictal metabolism. One of the first PET studies on patients with various epilepsy syndromes suggested that it may obviate the need for intracranial evaluation (182). This too optimistic view of the technique already revealed its potential usefulness especially in unclear clinical situations. FDG has a half-life of ca. 2 h and patients are observed before and after injection, ideally for 30–45 min. Then data is acquired for ca. 40 min, so the image represents the average glucose consumption over that time, with the earlier phase weighing more into the image. Ideally, EEG should be monitored during the “consumption period” (and even longer before) to avoid false “isometabolic” pattern (for instance, the result of a hypometabolic interictal pattern plus hypermetabolic pattern due to subclinical seizures). The spatial resolution of the technique is in the order of 4–8 mm, but the images should be viewed side-by-side with the subject MRI, or best, co-registered with MRI. In some centers, newer systems allowing single-session or even simultaneous acquisition of PET and MRI (183). Visual inspection is usually performed to interpret results in the clinical context, while statistical analyses are mostly used for group analysis. Statistical analysis seemed to improve the yield of FDG-PET in patients with extra-temporal lobe epilepsy compared to patients with temporal lobe epilepsy, increasing the sensitivity from 19–38 to 67% (184).

In surgical candidates with unilateral temporal lobe epilepsy, PET showed clearly visible unilateral hypometabolism, independently of the presence of a MRI abnormality (185). The same study showed that patients with PET hypometabolism concordant with EEG findings benefit from surgery as much as patients with hippocampal sclerosis identified on MRI: around 80% of them were seizure-free after 38 months of follow-up.

Focal interictal hypometabolism of FDG-PET is usually larger than the epileptogenic cortex, reflecting probably the altered function not only of the ictal focus, but also of the areas involved by the first ictal spread (186). It has been shown that the extent of resection of the hypometabolism correlates to outcome of temporal lobectomy (187). The assumption “more resection = better outcome” may well be true when considering only post-operative seizure outcomes in epileptic patients, but it is questionable in terms of cognitive and neurological outcome.

The mechanisms underlying the hypometabolism in epilepogenic cortex are still mostly unresolved: it is known that FDG-PET distribution reflects mainly synaptic activity, rather than cellular loss (188). It has been hypothesized that repeated seizures or dysfunctional cortex (like dysplasia or tubers) induce a protective inhibitory effect through synaptic plasticity (189). This hypothesis is corroborated by the fact that reduction in glucose metabolism is related to epilepsy duration (190). Also, metabolism is affected by anti-epileptic drugs: patients on GABAergic drugs (e.g., benzodiazepines, barbiturates) often exhibit a diffuse hypometabolism which may hinder the identification of discrete areas of the true epileptogenic hypometabolism (191).

Several studies in patients with antero-mesial TLE have shown that hypometabolism can be found not only in the affected area, but also, to a lesser extent, ipsilaterally in the frontal, parietal, insular cortex, thalamus, and basal ganglia (186), corroborating the concept of epileptic network. In unilateral mesial temporal lobe epilepsy bilateral temporal lobe hypometabolism can often be detected, causing ambiguity in lateralizing the epileptogenic zone. It has been shown that having had a recent seizure is the major factor related to this situation: bilateral temporal lobe hypometabolism can be avoided by performing PET scan more than 2 days after the last seizure (192).

Concerning extra-temporal lobe epilepsy, the role of PET is promising, but less well established. A retrospective study on 23 patients with MRI-negative focal cortical dysplasia, who then underwent to surgery, has shown focal or regional concordant PET abnormalities in 22/23 cases, detected either by visual analysis alone or PET/MRI co-registration (193). Twenty of 23 patients were seizure-free at 4 years after a limited areas resection that showed a Taylor-type focal cortical dysplasia in all cases. Another study on 14 patients with similar clinical characteristics has shown that the complete resection of the dysplastic cortex localized by FDG-PET, SISCOM, or intracranial EEG is a reliable predictor of favorable post-operative outcome (194). Therefore, in extra-temporal lobe epilepsies, PET is best used as a guide for focusing the review of MRI in the search for subtle overlooked cortical dysplasia or to inform the placement of intracranial electrodes.

Various other tracers potentially useful in the setting of pre-surgical evaluation are currently limited to few centers and mostly for research purposes, as most of them are based on radiolabeled carbon whose production and use is more difficult than FDG. These are either ligands of specific receptors or neurotransmitters precursor, or transporters, like GABA, glutamate, serotonin, adenosine, acetylcholine, and opioid system. Among these, alpha-methyl-tryptophan has been shown to be superior to FDG for identifying the epileptogenic tuber (195) in a population of seventeen operated children with tuberous sclerosis. The GABA antagonist flumazenil (FMZ) seems to be more sensitive than FDG for lateralizing the focus in temporal lobe epilepsy (196). Increased periventricular [11C] FMZ binding, reflecting heterotopic neuron concentration, has been described as one predictor of bad outcome in patients operated for hippocampal sclerosis (197).

## Single Photon Emission Computed Tomography

Sir Victor Horsley was the first to notice directly during brain surgery that cortical blood flow increases during a seizure more than 100 years ago (198). SPECT imaging uses tracers, like 99mTc-labeled compounds [hexamethylpropylenamine oxime (HMPAO) or ethyl cysteinate dimer (ECD)] (mainly used in our work-up) that freely cross the blood brain barrier, providing, in this way, information about cerebral blood flow (199). The tracer distributes rapidly and then their distribution is stable for some hours. So, even if acquired a few hours from the injection (time sometimes necessary to move the patient in the SPECT scanner), the images reflect the focal increase of perfusion at the moment of the seizure. If given during the interictal period, reduced or normal uptake can be observed, thus results are ambiguous regarding focus localization. It is important to perform interictal SPECT so that a baseline is available to compare the ictal results.

The SPECT procedure requires expert and vigilant video-EEG monitoring to determine the presence of a seizure. Within a few seconds, the staff needs to read the EEG and/or identifies the patient’s habitual seizures, and inject the tracer. Analysis should be done by comparing the ictal with the interictal exam, either by visual analysis or by computer-aided algorithms, like SISCOM (Subtraction of ictal SPECT co-registered to MRI) (200). The area of maximal perfusion change during the seizure is then co-registered to the patient’s MRI.

It has been shown that in temporal lobe epilepsy the initial hyperperfusion of the seizure-onset zone and the propagating areas is followed by a hypoperfusion of the same areas, probably because of an auto-regulatory mechanism (201). Indeed there is a gradual change from the hyper- to the hypoperfused state of the focus with adjacent hypoperfusion, which evolves toward an isoperfused state and gradual recovery in the adjacent regions (202). Depending on the time of injection, SPECT images can reflect the seizure-onset zone, or the propagation areas (203). If the tracer is injected more than 20 s after the onset of the seizure, the precise localization can no more reliably be determined (204, 205). Careful review of the seizure video during which the SPECT was carried out is important to verify ictal injection. Another important concept is that, the accuracy of SPECT in the evaluation of short seizures (i.e., <20 s) is much less precise, as 10–20 s are necessary to transport the tracer to the brain.

Different studies have addressed the yield of (ictal SPECT), or have compared it with other pre-surgical techniques.

An extensive review of 39 SPECT studies found that ictal SPECT was correct in 70–100% of patients with temporal lobe epilepsy, suggesting that the technique has a better localization yield in this type of epilepsy (206). Nevertheless, a later study has shown an equivalent or even better yield for extra-temporal epilepsy [86% in extra-temporal compared to 67% in temporal lobe epilepsy (207)]. Some studies that included specifically patients with non-lesional extra-temporal epilepsy have shown that the resection of the SISCOM area is related to good post-operative outcome (194, 208). Similar findings have been corroborated by a prospective study (209), which has shown that if SISCOM is concordant with the future resection site, there are higher chances of having good to excellent post-operative outcome. A study on 71 patients with non-lesional temporal epilepsy with a follow-up of >2 years has shown that SPECT is less performant than PET (210): the sensitivity of ictal SPECT was 76% (vs. 37% for the interictal SPECT) and specificity was only 25%. Nevertheless, in patients with intracranial EEG validation of the seizure-onset zone, SPECT/SISCOM appeared to be more sensitive than PET, providing new, and complementary information (211). Our experience (39), using post-operative outcome as gold standard, suggests grossly comparable sensitivity between PET and SPECT (PET: 68%, ictal SPECT/SISCOM: 57%) as well as for sensitivity (PET: 44%, ictal SPECT/SISCOM: 47%).

## Magnetic Resonance Spectroscopy

Magnetic resonance spectroscopy is a non-invasive technique that maps brain metabolism by measuring concentrations of metabolites and neurotransmitters in the brain tissue. Reduced neuronal markers (*N*-acetyl-aspartate) and increased glial markers (choline) are compatible with focal epileptogenic lesion; lactate increase suggests the presence of epileptic activity, which can have local and remote effects, even on contralateral structures (212).

Whereas studies in temporal and extra-temporal epilepsies (some of them with post-operative validation) have showed multifocal metabolic changes without reliable lateralization or localization value (213–215), a recent study suggests that spectroscopy might have a higher predictive value at 7T (216).

## Multimodal Imaging, Co-Registration and Perspectives

**Figure 2:**
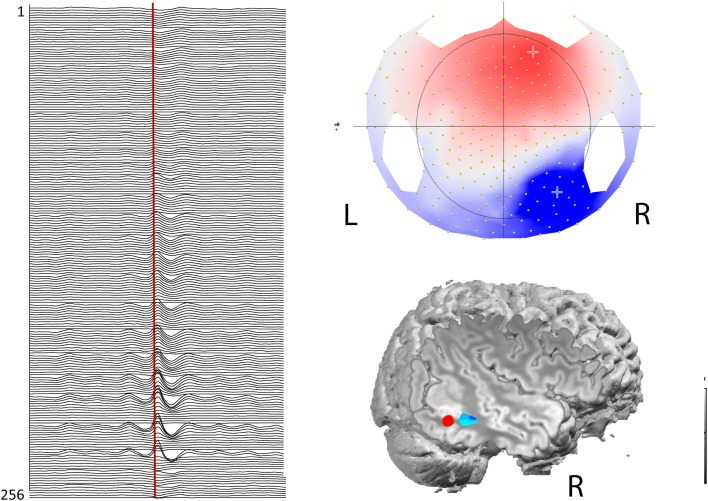
**EEG-fMRI and ESI in a patient with right posterior quadrant epilepsy and temporo-parieto-occipital heterotopia**. On the left: averaged spikes with equipotential at T8-P8 in a 256 channels EEG recording (voltage map: on the right superior corner). Right inferior corner: co-registration between the patient’s MRI, ESI (in red) and BOLD response (in blue) to the same type of spikes, recorded in a separate EEG-fMRI session. Both ESI and EEG-fMRI show a maximum value in the same part of the lesion. ESI was computed at the 50% of arising phase of the spike.

The combination of functional techniques can yield complementary information. For instance, EEG-fMRI and ESI measure different signals with different time scale: electrical signal is in the milliseconds order, whereas metabolic response is in the seconds order. In most patients with focal epilepsy, part of the BOLD response to IEDs is highly concordant with ESI (Figure 2) even when the two techniques were applied subsequently (217). ESI performed during fMRI recordings allows distinguishing between hemodynamic changes related to spike onset vs. propagation, giving important complementary information to the limited fMRI temporal resolution (125). A study investigating the possibility of using electric source time course for guiding fMRI analysis found that this solution could improve EEG-fMRI analysis. This strategy represents some spatial and temporal filter of EEG-fMRI based on ESI (127). A similar approach improves the interpretation of IED-associated networks of BOLD changes in pediatric focal epilepsies and in epileptic encephalopathy with continuous spikes and waves during slow sleep (123, 126, 218). Such validation of topographic analysis of EEG during fMRI supports the use of advanced EEG features for guiding fMRI analysis. Likewise, BOLD-correlates of pathological EEG topographies markedly improved the sensitivity and specificity of EEG-fMRI, even in the absence of IED (Figure 1) (137, 139, 219). It could be wondered how reliable is ESI when performed on pre-processed in-scanner EEG data. ESI outside and inside the scanner is reportedly unchanged but no formal comparison is available. A current problem is that ESI and EEG-fMRI acquisitions and analysis change a lot across centers (220), so comparison among centers could be difficult. Guidelines or recommendations on state of the art of these techniques would be useful in the future.

Integrating these studies with simultaneous scalp and intracranial EEG (52) as well as simultaneous intracranial EEG and fMRI (179, 180) will help address some important issues in the field, such as the ability of ESI to detect mesial temporal activity and important aspects of the neuro-vascular coupling. This is a crucial topic in order to understand mechanisms of EEG-fMRI as it has been reported that electro-physiological and fMRI maps have intrinsic spatial differences (221).

The combination between functional connectivity and structural connectivity, revealed by MRI tractography, could inform on direct and indirect connections within these networks (222, 223).

The understanding of the etiopathogenesis of epileptic syndromes, particularly those with unknown causes, can be provided by PET and SPECT, by revealing various underlying abnormalities that may not be fully appreciated from MR imaging studies (224). The yield of the different combinations of techniques might depend on the localization and etiology. For instance, in tuberous sclerosis, the combination of ESI and PET had a higher yield for localizing the epileptic tuber than the combination of SPECT with either technique (225).

**Figure 3:**
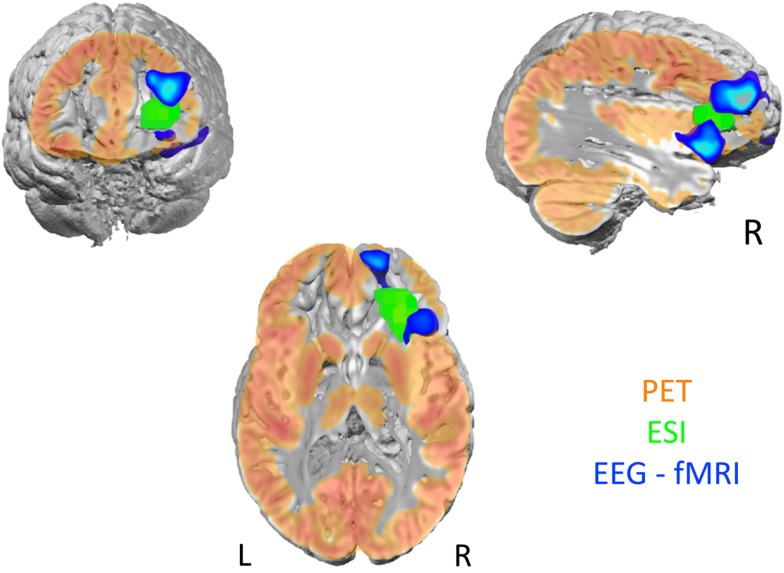
**Multimodal functional imaging approach to localize the epileptic focus in a patient with right orbito-frontal focal cortical dysplasia**. The hypometabolic region detected by PET (orange) contains both the ESI (green; 256 electrodes, lSMAC, LORETA) and the IED related BOLD response (blue; marked events: FP2-F8 spikes).

**Figure 4:**
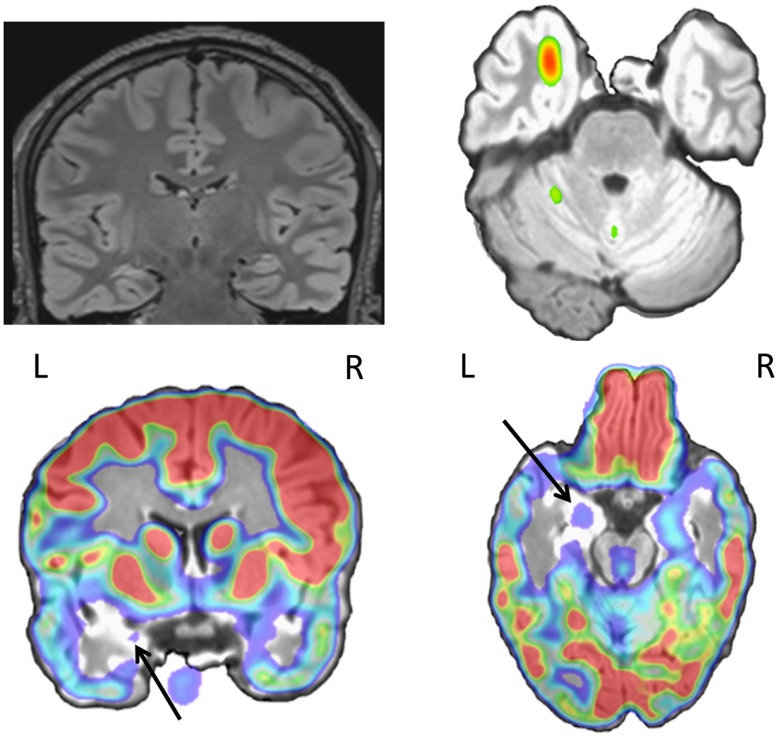
**Twenty-seven-year-old patient with left mesial temporal lobe sclerosis (FLAIR sequence, coronal view) and bitemporal interictal spikes**. PET showed hypometabolism on the left mesial temporal structures and temporal pole (black arrow). SISCOM showed concordant ictal hyperperfusion (maximum in the temporal pole, shown in the figure).

Beyond clinical MRI and EEG, the pre-surgical work-up shows a high variability across centers depending on the accessible technology and local expertise in structural functional and multimodal imaging. However, a broadly recognized common point is that concordance between the different tests (Figures 3 and 4) is crucial for guiding clinical decision. Multimodal functional imaging offers a more detailed picture of the brain networks involved in epileptic activity in individual patients. As a consequence, surgery with or without intracranial recordings can be offered to increasingly difficult cases, while multimodal concordance increases the chances of favorable outcome (226). A precise co-registration between modalities, with alignment of structural and fMRI, isotopic imaging, electro-physiological sources and high resolution structural MRI for anatomical localization, is obligatory to assess concordance between modalities and guarantee precise intracranial electrode placement and resection margins based on the imaging findings. Co-registration of post-implantation imaging is useful to validate the placement of intracranial electrodes and co-registration with post-operative images. The outline of the performed resection will reveal whether the targeted brain structures and lesions have been entirely removed. In this process, it is important that brain deformation and distortions specific to each image modality are taken into account. Automatization and reduced processing time now allow considering the integration of multimodal preoperative data into the neuronavigation suite for assisting intra-operative decision-making.

## Conclusion

Multimodal imaging and future developments of neuroimaging techniques improve our understanding of the dynamics of brain with high spatial and temporal resolutions. The detection of subtle structural or functional abnormalities allow considering surgery in a greater number of difficult cases. Imaging findings have a role in guiding the implantation of intracranial electrodes, for improving the success of subsequent surgery. Hopefully, more patients will benefit from surgery without the need for invasive recordings. The concordance between different imaging techniques facilitates better mapping of epileptic zone, epileptic networks, and eloquent cortices. The understanding of functional brain networks will allow us to better understand the neurobiology of epilepsies and develop new diagnostic, prognostic and therapeutic tools.

## Conflict of Interest Statement

The authors declare that the research was conducted in the absence of any commercial or financial relationships that could be construed as a potential conflict of interest.

## References

[1] Sander JW The epidemiology of epilepsy revisited. Curr Opin Neurol (2003) 16:165–70 10.1097/00019052-200304000-00008 12644744

[2] Hauser WA Annegers JF Kurland LT Incidence of epilepsy and unprovoked seizures in Rochester, Minnesota: 1935–1984. Epilepsia (1993) 34:453–68 8504780 10.1111/j.1528-1157.1993.tb02586.x

[3] Kwan P Sander JW The natural history of epilepsy: an epidemiological view. J Neurol Neurosurg Psychiatry (2004) 75:1376–81 10.1136/jnnp.2004.045690 15377680 PMC1738749

[4] Schuele SU Luders HO Intractable epilepsy: management and therapeutic alternatives. Lancet Neurol (2008) 7:514–24 10.1016/S1474-4422(08)70108-X 18485315

[5] Brodie MJ Barry SJ Bamagous GA Norrie JD Kwan P Patterns of treatment response in newly diagnosed epilepsy. Neurology (2012) 78:1548–54 10.1212/WNL.0b013e3182563b19 22573629 PMC3348850

[6] Bancaud J Topographic relationship between cerebral lesions and seizures discharges. In: Canger R Angeleri F Penry JK, editors. Advances in Epileptology, XIth Epilepsy International Symposium New York: Raven Press (1980). p. 103–9

[7] Spencer S Huh L Outcomes of epilepsy surgery in adults and children. Lancet Neurol (2008) 7:525–37 10.1016/S1474-4422(08)70109-1 18485316

[8] de Tisi J Bell GS Peacock JL Mcevoy AW Harkness WF Sander JW The long-term outcome of adult epilepsy surgery, patterns of seizure remission, and relapse: a cohort study. Lancet (2011) 378:1388–95 10.1016/S0140-6736(11)60890-8 22000136

[9] Wiebe S Blume WT Girvin JP Eliasziw M Effectiveness, and Efficiency of Surgery for Temporal Lobe Epilepsy Study G A randomized, controlled trial of surgery for temporal-lobe epilepsy. N Engl J Med (2001) 345:311–8 10.1056/NEJM200108023450501 11484687

[10] Engel J Jr Wiebe S French J Sperling M Williamson P Spencer D Practice parameter: temporal lobe and localized neocortical resections for epilepsy: report of the Quality Standards Subcommittee of the American Academy of Neurology, in association with the American Epilepsy Society and the American Association of Neurological Surgeons. Neurology (2003) 60:538–47 10.1212/01.WNL.0000055086.35806.2 12601090

[11] Hakimi AS Spanaki MV Schuh LA Smith BJ Schultz L A survey of neurologists’ views on epilepsy surgery and medically refractory epilepsy. Epilepsy Behav (2008) 13:96–101 10.1016/j.yebeh.2008.02.003 18337180

[12] De Flon P Kumlien E Reuterwall C Mattsson P Empirical evidence of underutilization of referrals for epilepsy surgery evaluation. Eur J Neurol (2010) 17:619–25 10.1111/j.1468-1331.2009.02891.x 20039934

[13] Haneef Z Stern J Dewar S Engel J Jr Referral pattern for epilepsy surgery after evidence-based recommendations: a retrospective study. Neurology (2010) 75:699–704 10.1212/WNL.0b013e3181eee457 20733145 PMC2931651

[14] Hader WJ Tellez-Zenteno J Metcalfe A Hernandez-Ronquillo L Wiebe S Kwon CS Complications of epilepsy surgery: a systematic review of focal surgical resections and invasive EEG monitoring. Epilepsia (2013) 54:840–7 10.1111/epi.12161 23551133

[15] Neligan A Bell GS Shorvon SD Sander JW Temporal trends in the mortality of people with epilepsy: a review. Epilepsia (2010) 51:2241–6 10.1111/j.1528-1167.2010.02711.x 21175603

[16] Sillanpaa M Shinnar S Long-term mortality in childhood-onset epilepsy N Engl J Med (2010) 363:2522–9 10.1056/NEJMoa0911610 21175314

[17] Kwan P Arzimanoglou A Berg AT Brodie MJ Allen Hauser W Mathern G Definition of drug resistant epilepsy: consensus proposal by the ad hoc Task Force of the ILAE Commission on Therapeutic Strategies. Epilepsia (2010) 51:1069–77 10.1111/j.1528-1167.2009.02397.x 19889013

[18] Rosenow F Luders H Presurgical evaluation of epilepsy. Brain (2001) 124:1683–700 10.1093/brain/124.9.1683 11522572

[19] Cossu M Cardinale F Castana L Citterio A Francione S Tassi L Stereoelectroencephalography in the presurgical evaluation of focal epilepsy: a retrospective analysis of 215 procedures. Neurosurgery (2005) 57:706–18 10.1227/01.NEU.0000176656.33523.1e discussion 706-718, 16239883

[20] Cossu M Chabardes S Hoffmann D Lo Russo G [Presurgical evaluation of intractable epilepsy using stereo-electro-encephalography methodology: principles, technique and morbidity]. Neurochirurgie (2008) 54:367–73 10.1016/j.neuchi.2008.02.031 18440035

[21] Zumsteg D Wieser HG Presurgical evaluation: current role of invasive EEG. Epilepsia (2000) 41(Suppl 3):S55–60 10.1111/j.1528-1157.2000.tb01535.x 11001337

[22] Lehmann D Multichannel topography of human alpha EEG fields. Electroencephalogr Clin Neurophysiol (1971) 31:439–49 10.1016/0013-4694(71)90165-9 4107798

[23] He B Lian J High-resolution spatio-temporal functional neuroimaging of brain activity. Crit Rev Biomed Eng (2002) 30:283–306 10.1615/CritRevBiomedEng.v30.i456.30 12739752

[24] Michel CM Murray MM Lantz G Gonzalez S Spinelli L Grave De Peralta R EEG source imaging. Clin Neurophysiol (2004) 115:2195–222 10.1016/j.clinph.2004.06.001 15351361

[25] Michel CM He B EEG mapping and source imaging. In: Schomer D Lopes da Silva F, editors. Niedermeyer’s Electroencephalography. 6th ed Philadelphia, PA: Lippincott Williams & Wilkins (2011). p. 1179–202

[26] Lantz G Michel CM Seeck M Blanke O Spinelli L Thut G Space-oriented segmentation and 3-dimensional source reconstruction of ictal EEG patterns. Clin Neurophysiol (2001) 112:688–97 10.1016/S1388-2457(01)00479-5 11275543

[27] Michel CM Lantz G Spinelli L De Peralta RG Landis T Seeck M 128-channel EEG source imaging in epilepsy: clinical yield and localization precision. J Clin Neurophysiol (2004) 21:71–83 10.1097/00004691-200403000-00001 15284597

[28] Plummer C Harvey AS Cook M EEG source localization in focal epilepsy: where are we now? Epilepsia (2008) 49:201–18 10.1111/j.1528-1167.2007.01381.x 17941844

[29] Ebersole JS Hawes-Ebersole S Clinical application of dipole models in the localization of epileptiform activity. J Clin Neurophysiol (2007) 24:120–9 10.1097/WNP.0b013e31803ece13 17414967

[30] Spinelli L Andino SG Lantz G Seeck M Michel CM Electromagnetic inverse solutions in anatomically constrained spherical head models. Brain Topogr (2000) 13:115–25 10.1023/A:1026607118642 11154101

[31] Hallez H Vanrumste B Grech R Muscat J De Clercq W Vergult A Review on solving the forward problem in EEG source analysis. J Neuroeng Rehabil (2007) 4:46 10.1186/1743-0003-4-46 18053144 PMC2234413

[32] Brodbeck V Lascano AM Spinelli L Seeck M Michel CM Accuracy of EEG source imaging of epileptic spikes in patients with large brain lesions. Clin Neurophysiol (2009) 120:679–85 10.1016/j.clinph.2009.01.011 19264547

[33] Alarcon G Guy CN Binnie CD Walker SR Elwes RD Polkey CE Intracerebral propagation of interictal activity in partial epilepsy: implications for source localisation. J Neurol Neurosurg Psychiatry (1994) 57:435–49 10.1136/jnnp.57.4.435 8163992 PMC1072872

[34] Lantz G Spinelli L Seeck M De Peralta Menendez RG Sottas CC Michel CM Propagation of interictal epileptiform activity can lead to erroneous source localizations: a 128-channel EEG mapping study. J Clin Neurophysiol (2003) 20:311–9 10.1097/00004691-200309000-00003 14701992

[35] Chowdhury RA Lina JM Kobayashi E Grova C MEG source localization of spatially extended generators of epileptic activity: comparing entropic and hierarchical bayesian approaches. PLoS One (2013) 8:e55969 10.1371/journal.pone.0055969 23418485 PMC3572141

[36] Kaiboriboon K Luders HO Hamaneh M Turnbull J Lhatoo SD EEG source imaging in epilepsy – practicalities and pitfalls. Nat Rev Neurol (2012) 8:498–507 10.1038/nrneurol.2012.150 22868868

[37] Lantz G Grave De Peralta R Spinelli L Seeck M Michel CM Epileptic source localization with high density EEG: how many electrodes are needed? Clin Neurophysiol (2003) 114:63–9 10.1016/S1388-2457(02)00337-1 12495765

[38] Sperli F Spinelli L Seeck M Kurian M Michel CM Lantz G EEG source imaging in pediatric epilepsy surgery: a new perspective in presurgical workup. Epilepsia (2006) 47:981–90 10.1111/j.1528-1167.2006.00550.x 16822244

[39] Brodbeck V Spinelli L Lascano AM Wissmeier M Vargas MI Vulliemoz S Electroencephalographic source imaging: a prospective study of 152 operated epileptic patients. Brain (2011) 134:2887–97 10.1093/brain/awr243 21975586 PMC3187544

[40] Ebersole JS Wade PB Spike voltage topography and equivalent dipole localization in complex partial epilepsy. Brain Topogr (1990) 3:21–34 10.1007/BF01128858 2094309

[41] Hufnagel A Dumpelmann M Zentner J Schijns O Elger CE Clinical relevance of quantified intracranial interictal spike activity in presurgical evaluation of epilepsy. Epilepsia (2000) 41:467–78 10.1111/j.1528-1157.2000.tb00191.x 10756415

[42] Brodbeck V Spinelli L Lascano AM Pollo C Schaller K Vargas MI Electrical source imaging for presurgical focus localization in epilepsy patients with normal MRI. Epilepsia (2010) 51:583–91 10.1111/j.1528-1167.2010.02521.x 20196796

[43] Gavaret M Guedj E Koessler L Trebuchon-Da Fonseca A Aubert S Mundler O Reading epilepsy from the dominant temporo-occipital region. J Neurol Neurosurg Psychiatry (2010) 81:710–5 10.1136/jnnp.2009.175935 19620139

[44] Noe K Sulc V Wong-Kisiel L Wirrell E Van Gompel JJ Wetjen N Long-term outcomes after nonlesional extratemporal lobe epilepsy surgery. JAMA Neurol (2013) 70:1003–8 10.1001/jamaneurol.2013.209 23732844 PMC3920594

[45] Mégevand P Spinelli L Genetti M Brodbeck V Momjian S Schaller K Electric source imaging of interictal activity accurately localises the seizure onset zone. J Neurol Neurosurg Psychiatry (2014) 85:38–43 10.1136/jnnp-2013-305515 23899624

[46] Gavaret M Badier JM Marquis P Bartolomei F Chauvel P Electric source imaging in temporal lobe epilepsy. J Clin Neurophysiol (2004) 21:267–82 10.1097/01.WNP.0000139929.06455.82 15509916

[47] Gavaret M Badier JM Marquis P Mcgonigal A Bartolomei F Regis J Electric source imaging in frontal lobe epilepsy. J Clin Neurophysiol (2006) 23:358–70 10.1097/01.wnp.0000214588.94843.c2 16885710

[48] Nayak D Valentin A Alarcon G Garcia Seoane JJ Brunnhuber F Juler J Characteristics of scalp electrical fields associated with deep medial temporal epileptiform discharges. Clin Neurophysiol (2004) 115:1423–35 10.1016/j.clinph.2004.01.009 15134711

[49] Zumsteg D Friedman A Wennberg RA Wieser HG Source localization of mesial temporal interictal epileptiform discharges: correlation with intracranial foramen ovale electrode recordings. Clin Neurophysiol (2005) 116:2810–8 10.1016/j.clinph.2005.08.009 16253551

[50] James CE Britz J Vuilleumier P Hauert CA Michel CM Early neuronal responses in right limbic structures mediate harmony incongruity processing in musical experts. Neuroimage (2008) 42:1597–608 10.1016/j.neuroimage.2008.06.025 18640279

[51] Nahum L Gabriel D Spinelli L Momjian S Seeck M Michel CM Rapid consolidation and the human hippocampus: intracranial recordings confirm surface EEG. Hippocampus (2011) 21:689–93 10.1002/hipo.20819 20865742

[52] Yamazaki M Tucker DM Fujimoto A Yamazoe T Okanishi T Yokota T Comparison of dense array EEG with simultaneous intracranial EEG for interictal spike detection and localization. Epilepsy Res (2012) 98:166–73 10.1016/j.eplepsyres.2011.09.007 22018998

[53] Gavaret M Trebuchon A Bartolomei F Marquis P Mcgonigal A Wendling F Source localization of scalp-EEG interictal spikes in posterior cortex epilepsies investigated by HR-EEG and SEEG. Epilepsia (2009) 50:276–89 10.1111/j.1528-1167.2008.01742.x 18717708

[54] Holmes MD Tucker DM Quiring JM Hakimian S Miller JW Ojemann JG Comparing noninvasive dense array and intracranial electroencephalography for localization of seizures. Neurosurgery (2010) 66:354–62 10.1227/01.NEU.0000363721.06177.07 20087136

[55] Koessler L Benar C Maillard L Badier JM Vignal JP Bartolomei F Source localization of ictal epileptic activity investigated by high resolution EEG and validated by SEEG. Neuroimage (2010) 51:642–53 10.1016/j.neuroimage.2010.02.067 20206700

[56] Yang L Wilke C Brinkmann B Worrell GA He B Dynamic imaging of ictal oscillations using non-invasive high-resolution EEG. Neuroimage (2011) 56:1908–17 10.1016/j.neuroimage.2011.03.043 21453776 PMC3359824

[57] Holmes MD Brown M Tucker DM Saneto RP Miller KJ Wig GS Localization of extratemporal seizure with noninvasive dense-array EEG. Comparison with intracranial recordings. Pediatr Neurosurg (2008) 44:474–9 10.1159/000180302 19066438

[58] Blanke O Lantz G Seeck M Spinelli L Grave De Peralta R Thut G Temporal and spatial determination of EEG-seizure onset in the frequency domain. Clin Neurophysiol (2000) 111:763–72 10.1016/S1388-2457(00)00251-0 10802445

[59] Assaf BA Ebersole JS Visual and quantitative ictal EEG predictors of outcome after temporal lobectomy. Epilepsia (1999) 40:52–61 10.1111/j.1528-1157.1999.tb01988.x 9924902

[60] Merlet I Gotman J Dipole modeling of scalp electroencephalogram epileptic discharges: correlation with intracerebral fields. Clin Neurophysiol (2001) 112:414–30 10.1016/S1388-2457(01)00458-8 11222962

[61] Boon P D’Have M Vanrumste B Van Hoey G Vonck K Van Walleghem P Ictal source localization in presurgical patients with refractory epilepsy. J Clin Neurophysiol (2002) 19:461–8 10.1097/00004691-200210000-00009 12477991

[62] Lu Y Yang L Worrell GA He B Seizure source imaging by means of FINE spatio-temporal dipole localization and directed transfer function in partial epilepsy patients. Clin Neurophysiol (2012) 123:1275–83 10.1016/j.clinph.2011.11.007 22172768 PMC3327757

[63] Hamalainen MS Hari R Ilmoniemi RJ Knuutila J Lounesmaa OV Magneto-encephalography: theory, instrumentation, and applications to noninvasive studies of the working human brain. Rev Mod Phys (1993) 65:413–97 10.1103/RevModPhys.65.413

[64] Murakami S Okada Y Contributions of principal neocortical neurons to magnetoencephalography and electroencephalography signals. J Physiol (2006) 575:925–36 10.1113/jphysiol.2006.105379 16613883 PMC1995687

[65] Malmivuo J Comparison of the properties of EEG and MEG in detecting the electric activity of the brain. Brain Topogr (2012) 25:1–19 10.1007/s10548-011-0202-1 21912974

[66] Lopes da Silva F EEG and MEG: relevance to neuroscience. Neuron (2013) 80:1112–28 10.1016/j.neuron.2013.10.017 24314724

[67] Ahlfors SP Han J Belliveau JW Hamalainen MS Sensitivity of MEG and EEG to source orientation. Brain Topogr (2010) 23:227–32 10.1007/s10548-010-0154-x 20640882 PMC2914866

[68] Knowlton RC Razdan SN Limdi N Elgavish RA Killen J Blount J Effect of epilepsy magnetic source imaging on intracranial electrode placement. Ann Neurol (2009) 65:716–23 10.1002/ana.21660 19557860 PMC2729691

[69] Sutherling WW Mamelak AN Thyerlei D Maleeva T Minazad Y Philpott L Influence of magnetic source imaging for planning intracranial EEG in epilepsy. Neurology (2008) 71:990–6 10.1212/01.wnl.0000326591.29858.1a 18809834 PMC2676955

[70] Bouet R Jung J Delpuech C Ryvlin P Isnard J Guenot M Towards source volume estimation of interictal spikes in focal epilepsy using magnetoencephalography. Neuroimage (2012) 59:3955–66 10.1016/j.neuroimage.2011.10.052 22036998

[71] Jung J Bouet R Delpuech C Ryvlin P Isnard J Guenot M The value of magnetoencephalography for seizure-onset zone localization in magnetic resonance imaging-negative partial epilepsy. Brain (2013) 136:3176–86 10.1093/brain/awt213 24014520 PMC3784280

[72] Fischer MJ Scheler G Stefan H Utilization of magnetoencephalography results to obtain favourable outcomes in epilepsy surgery. Brain (2005) 128:153–7 10.1093/brain/awh333 15563514

[73] Genow A Hummel C Scheler G Hopfengartner R Kaltenhauser M Buchfelder M Epilepsy surgery, resection volume and MSI localization in lesional frontal lobe epilepsy. Neuroimage (2004) 21:444–9 10.1016/j.neuroimage.2003.08.029 14741681

[74] Agirre-Arrizubieta Z Huiskamp GJ Ferrier CH Van Huffelen AC Leijten FS Interictal magnetoencephalography and the irritative zone in the electrocorticogram. Brain (2009) 132:3060–71 10.1093/brain/awp137 19498089

[75] Knowlton RC Elgavish RA Bartolucci A Ojha B Limdi N Blount J Functional imaging: II. Prediction of epilepsy surgery outcome. Ann Neurol (2008) 64:35–41 10.1002/ana.21419 18570291

[76] Oishi M Otsubo H Kameyama S Morota N Masuda H Kitayama M Epileptic spikes: magnetoencephalography versus simultaneous electrocorticography. Epilepsia (2002) 43:1390–5 10.1046/j.1528-1157.2002.10702.x 12423390

[77] Huiskamp G Agirre-Arrizubieta Z Leijten F Regional differences in the sensitivity of MEG for interictal spikes in epilepsy. Brain Topogr (2010) 23:159–64 10.1007/s10548-010-0134-1 20151193 PMC2874057

[78] Santiuste M Nowak R Russi A Tarancon T Oliver B Ayats E Simultaneous magnetoencephalography and intracranial EEG registration: technical and clinical aspects. J Clin Neurophysiol (2008) 25:331–9 10.1097/WNP.0b013e31818e7913 18997623

[79] Fujiwara H Greiner HM Hemasilpin N Lee KH Holland-Bouley K Arthur T Ictal MEG onset source localization compared to intracranial EEG and outcome: improved epilepsy presurgical evaluation in pediatrics. Epilepsy Res (2012) 99:214–24 10.1016/j.eplepsyres.2011.11.007 22178034 PMC3520066

[80] Ossenblok P De Munck JC Colon A Drolsbach W Boon P Magnetoencephalography is more successful for screening and localizing frontal lobe epilepsy than electroencephalography. Epilepsia (2007) 48:2139–49 10.1111/j.1528-1167.2007.01223.x 17662061

[81] Jansen FE Huiskamp G Van Huffelen AC Bourez-Swart M Boere E Gebbink T Identification of the epileptogenic tuber in patients with tuberous sclerosis: a comparison of high-resolution EEG and MEG. Epilepsia (2006) 47:108–14 10.1111/j.1528-1167.2006.00373.x 16417538

[82] Gavaret M Badier JM Bartolomei F Benar CG Chauvel P MEG and EEG sensitivity in a case of medial occipital epilepsy. Brain Topogr (2014) 27:192–6 10.1007/s10548-013-0317-7 24005334

[83] Jackson GD Connelly A Cross JH Gordon I Gadian DG Functional magnetic resonance imaging of focal seizures. Neurology (1994) 44:850–6 10.1212/WNL.44.5.850 8190287

[84] Krings T Topper R Reinges MH Foltys H Spetzger U Chiappa KH Hemodynamic changes in simple partial epilepsy: a functional MRI study. Neurology (2000) 54:524–7 10.1212/WNL.54.2.524 10668734

[85] Detre JA Sirven JI Alsop DC O’Connor MJ French JA Localization of subclinical ictal activity by functional magnetic resonance imaging: correlation with invasive monitoring. Ann Neurol (1995) 38:618–24 10.1002/ana.410380410 7574458

[86] Ives JR Warach S Schmitt F Edelman RR Schomer DL Monitoring the patient’s EEG during echo planar MRI. Electroencephalogr Clin Neurophysiol (1993) 87:417–20 10.1016/0013-4694(93)90156-P 7508375

[87] Gotman J Epileptic networks studied with EEG-fMRI. Epilepsia (2008) 49(Suppl 3):42–51 10.1111/j.1528-1167.2008.01509.x 18304255 PMC3792078

[88] Gotman J Kobayashi E Bagshaw AP Benar CG Dubeau F Combining EEG and fMRI: a multimodal tool for epilepsy research. J Magn Reson Imaging (2006) 23:906–20 10.1002/jmri.20577 16649203

[89] Ullsperger M Debener S Simultaneous EEG–fMRI: Recording, Analysis and Application. New York: Oxford University Press (2010).

[90] Krakow K Woermann FG Symms MR Allen PJ Lemieux L Barker GJ EEG-triggered functional MRI of interictal epileptiform activity in patients with partial seizures. Brain (1999) 122(Pt 9):1679–88 10.1093/brain/122.9.1679 10468507

[91] Seeck M Lazeyras F Michel CM Blanke O Gericke CA Ives J Non-invasive epileptic focus localization using EEG-triggered functional MRI and electromagnetic tomography. Electroencephalogr Clin Neurophysiol (1998) 106:508–12 10.1016/S0013-4694(98)00017-0 9741750

[92] Warach S Ives JR Schlaug G Patel MR Darby DG Thangaraj V EEG-triggered echo-planar functional MRI in epilepsy. Neurology (1996) 47:89–93 10.1212/WNL.47.1.89 8710131

[93] Allen PJ Josephs O Turner R A method for removing imaging artifact from continuous EEG recorded during functional MRI. Neuroimage (2000) 12:230–9 10.1006/nimg.2000.0599 10913328

[94] Bénar CG Gross DW Wang Y Petre V Pike B Dubeau F The BOLD response to interictal epileptiform discharges. Neuroimage (2002) 17:1182–92 10.1006/nimg.2002.1164 12414258

[95] Bénar CG Grova C Kobayashi E Bagshaw AP Aghakhani Y Dubeau F EEG-fMRI of epileptic spikes: concordance with EEG source localization and intracranial EEG. Neuroimage (2006) 30:1161–70 10.1016/j.neuroimage.2005.11.008 16413798

[96] Gholipour T Moeller F Pittau F Dubeau F Gotman J Reproducibility of interictal EEG-fMRI results in patients with epilepsy. Epilepsia (2011) 52:433–42 10.1111/j.1528-1167.2010.02768.x 21054351 PMC3792085

[97] Waites AB Shaw ME Briellmann RS Labate A Abbott DF Jackson GD How reliable are fMRI-EEG studies of epilepsy? A nonparametric approach to analysis validation and optimization. Neuroimage (2005) 24:192–9 10.1016/j.neuroimage.2004.09.005 15588610

[98] Flanagan D Abbott DF Jackson GD How wrong can we be? The effect of inaccurate mark-up of EEG/fMRI studies in epilepsy. Clin Neurophysiol (2009) 120:1637–47 10.1016/j.clinph.2009.04.025 19632890

[99] Liston AD De Munck JC Hamandi K Laufs H Ossenblok P Duncan JS Analysis of EEG-fMRI data in focal epilepsy based on automated spike classification and Signal Space Projection. Neuroimage (2006) 31:1015–24 10.1016/j.neuroimage.2006.01.040 16545967

[100] Kobayashi E Bagshaw AP Grova C Gotman J Dubeau F Grey matter heterotopia: what EEG-fMRI can tell us about epileptogenicity of neuronal migration disorders. Brain (2006) 129:366–74 10.1093/brain/awh710 16339793

[101] Tyvaert L Hawco C Kobayashi E Levan P Dubeau F Gotman J Different structures involved during ictal and interictal epileptic activity in malformations of cortical development: an EEG-fMRI study. Brain (2008) 131:2042–60 10.1093/brain/awn145 18669486 PMC3792088

[102] Moeller F Tyvaert L Nguyen DK Levan P Bouthillier A Kobayashi E EEG-fMRI: adding to standard evaluations of patients with nonlesional frontal lobe epilepsy. Neurology (2009) 73:2023–30 10.1212/WNL.0b013e3181c55d17 19996077 PMC2881856

[103] Thornton R Laufs H Rodionov R Cannadathu S Carmichael DW Vulliemoz S EEG correlated functional MRI and postoperative outcome in focal epilepsy. J Neurol Neurosurg Psychiatry (2010) 81:922–7 10.1136/jnnp.2009.196253 20547617

[104] An D Fahoum F Hall J Olivier A Gotman J Dubeau F Electroencephalography/functional magnetic resonance imaging responses help predict surgical outcome in focal epilepsy. Epilepsia (2013) 54:2184–94 10.1111/epi.12434 24304438 PMC4498907

[105] Thornton R Vulliemoz S Rodionov R Carmichael DW Chaudhary UJ Diehl B Epileptic networks in focal cortical dysplasia revealed using electroencephalography-functional magnetic resonance imaging. Ann Neurol (2011) 70:822–37 10.1002/ana.22535 22162063 PMC3500670

[106] Pittau F Dubeau F Gotman J Contribution of EEG/fMRI to the definition of the epileptic focus. Neurology (2012) 78:1479–87 10.1212/WNL.0b013e3182553bf7 22539574 PMC3345614

[107] Zijlmans M Huiskamp G Hersevoort M Seppenwoolde JH Van Huffelen AC Leijten FS EEG-fMRI in the preoperative work-up for epilepsy surgery. Brain (2007) 130:2343–53 10.1093/brain/awm141 17586868

[108] Archer JS Briellmann RS Syngeniotis A Abbott DF Jackson GD Spike-triggered fMRI in reading epilepsy: involvement of left frontal cortex working memory area. Neurology (2003) 60:415–21 10.1212/WNL.60.3.415 12578921

[109] Blauwblomme T Kahane P Minotti L Grouiller F Krainik A Vercueil L Multimodal imaging reveals the role of gamma activity in eating-reflex seizures. J Neurol Neurosurg Psychiatry (2011) 82:1171–3 10.1136/jnnp.2010.212696 21097547 PMC3338065

[110] Vaudano AE Carmichael DW Salek-Haddadi A Rampp S Stefan H Lemieux L Networks involved in seizure initiation. A reading epilepsy case studied with EEG-fMRI and MEG. Neurology (2012) 79:249–53 10.1212/WNL.0b013e31825fdf3a 22764255 PMC3398433

[111] Kobayashi E Bagshaw AP Benar CG Aghakhani Y Andermann F Dubeau F Temporal and extratemporal BOLD responses to temporal lobe interictal spikes. Epilepsia (2006) 47:343–54 10.1111/j.1528-1167.2006.00427.x 16499759

[112] Laufs H Hamandi K Salek-Haddadi A Kleinschmidt AK Duncan JS Lemieux L Temporal lobe interictal epileptic discharges affect cerebral activity in “default mode” brain regions. Hum Brain Mapp (2007) 28:1023–32 10.1002/hbm.20323 17133385 PMC2948427

[113] Varotto G Tassi L Franceschetti S Spreafico R Panzica F Epileptogenic networks of type II focal cortical dysplasia: a stereo-EEG study. Neuroimage (2012) 61:591–8 10.1016/j.neuroimage.2012.03.090 22510255

[114] Fahoum F Lopes R Pittau F Dubeau F Gotman J Widespread epileptic networks in focal epilepsies: EEG-fMRI study. Epilepsia (2012) 53:1618–27 10.1111/j.1528-1167.2012.03533.x 22691174 PMC4492710

[115] Engel J Jr Thompson PM Stern JM Staba RJ Bragin A Mody I Connectomics and epilepsy. Curr Opin Neurol (2013) 26:186–94 10.1097/WCO.0b013e32835ee5b8 23406911 PMC4064674

[116] Laufs H Richardson MP Salek-Haddadi A Vollmar C Duncan JS Gale K Converging PET and fMRI evidence for a common area involved in human focal epilepsies. Neurology (2011) 77:904–10 10.1212/WNL.0b013e31822c90f2 21849655 PMC3162638

[117] Flanagan D Badawy RA Jackson GD EEG-fMRI in focal epilepsy: Local activation and regional networks. Clin Neurophysiol (2014) 125:21–31 10.1016/j.clinph.2013.06.182 23871167

[118] Piredda S Gale K A crucial epileptogenic site in the deep prepiriform cortex. Nature (1985) 317:623–5 10.1038/317623a0 4058572

[119] Racine RJ Mosher M Kairiss EW The role of the pyriform cortex in the generation of interictal spikes in the kindled preparation. Brain Res (1988) 454:251–63 10.1016/0006-8993(88)90825-6 3409009

[120] Löscher W Ebert U The role of the piriform cortex in kindling. Prog Neurobiol (1996) 50:427–81 10.1016/S0301-0082(96)00036-6 9015822

[121] Kobayashi E Bagshaw AP Gotman J Dubeau F Metabolic correlates of epileptic spikes in cerebral cavernous angiomas. Epilepsy Res (2007) 73:98–103 10.1016/j.eplepsyres.2006.08.006 17000081

[122] Yu JM Tyvaert L Levan P Zelmann R Dubeau F Gotman J EEG spectral changes underlying BOLD responses contralateral to spikes in patients with focal epilepsy. Epilepsia (2009) 50:1804–9 10.1111/j.1528-1167.2009.02080.x 19389143

[123] Groening K Brodbeck V Moeller F Wolff S Van Baalen A Michel CM Combination of EEG-fMRI and EEG source analysis improves interpretation of spike-associated activation networks in paediatric pharmacoresistant focal epilepsies. Neuroimage (2009) 46:827–33 10.1016/j.neuroimage.2009.02.026 19264141

[124] Vulliemoz S Lemieux L Daunizeau J Michel CM Duncan JS The combination of EEG source imaging and EEG-correlated functional MRI to map epileptic networks. Epilepsia (2010) 51:491–505 10.1111/j.1528-1167.2009.02342.x 19817805

[125] Vulliemoz S Thornton R Rodionov R Carmichael DW Guye M Lhatoo S The spatio-temporal mapping of epileptic networks: combination of EEG-fMRI and EEG source imaging. Neuroimage (2009) 46:834–43 10.1016/j.neuroimage.2009.01.070 19408351 PMC2977852

[126] Siniatchkin M Groening K Moehring J Moeller F Boor R Brodbeck V Neuronal networks in children with continuous spikes and waves during slow sleep. Brain (2010) 133:2798–813 10.1093/brain/awq183 20688812

[127] Vulliemoz S Rodionov R Carmichael DW Thornton R Guye M Lhatoo SD Continuous EEG source imaging enhances analysis of EEG-fMRI in focal epilepsy. Neuroimage (2010) 49:3219–29 10.1016/j.neuroimage.2009.11.055 19948231

[128] Jacobs J Stich J Zahneisen B Asslander J Ramantani G Schulze-Bonhage A Fast fMRI provides high statistical power in the analysis of epileptic networks. Neuroimage (2014) 88:282–94 10.1016/j.neuroimage.2013.10.018 24140936

[129] Aghakhani Y Kobayashi E Bagshaw AP Hawco C Benar CG Dubeau F Cortical and thalamic fMRI responses in partial epilepsy with focal and bilateral synchronous spikes. Clin Neurophysiol (2006) 117:177–91 10.1016/j.clinph.2005.08.028 16314143

[130] Salek-Haddadi A Diehl B Hamandi K Merschhemke M Liston A Friston K Hemodynamic correlates of epileptiform discharges: an EEG-fMRI study of 63 patients with focal epilepsy. Brain Res (2006) 1088:148–66 10.1016/j.brainres.2006.02.098 16678803

[131] Lemieux L Salek-Haddadi A Lund TE Laufs H Carmichael D Modelling large motion events in fMRI studies of patients with epilepsy. Magn Reson Imaging (2007) 25:894–901 10.1016/j.mri.2007.03.009 17490845

[132] Liston AD Lund TE Salek-Haddadi A Hamandi K Friston KJ Lemieux L Modelling cardiac signal as a confound in EEG-fMRI and its application in focal epilepsy studies. Neuroimage (2006) 30:827–34 10.1016/j.neuroimage.2005.10.025 16343949

[133] Tyvaert L Levan P Grova C Dubeau F Gotman J Effects of fluctuating physiological rhythms during prolonged EEG-fMRI studies. Clin Neurophysiol (2008) 119:2762–74 10.1016/j.clinph.2008.07.284 18977169 PMC3792084

[134] Moehring J Coropceanu D Galka A Moeller F Wolff S Boor R Improving sensitivity of EEG-fMRI studies in epilepsy: the role of sleep-specific activity. Neurosci Lett (2011) 505:211–5 10.1016/j.neulet.2011.10.028 22027175

[135] Chaudhary UJ Rodionov R Carmichael DW Thornton RC Duncan JS Lemieux L Improving the sensitivity of EEG-fMRI studies of epileptic activity by modelling eye blinks, swallowing and other video-EEG detected physiological confounds. Neuroimage (2012) 61:1383–93 10.1016/j.neuroimage.2012.03.028 22450296

[136] Laufs H Hamandi K Walker MC Scott C Smith S Duncan JS EEG-fMRI mapping of asymmetrical delta activity in a patient with refractory epilepsy is concordant with the epileptogenic region determined by intracranial EEG. Magn Reson Imaging (2006) 24:367–71 10.1016/j.mri.2005.12.026 16677942

[137] Jann K Wiest R Hauf M Meyer K Boesch C Mathis J BOLD correlates of continuously fluctuating epileptic activity isolated by independent component analysis. Neuroimage (2008) 42:635–48 10.1016/j.neuroimage.2008.05.001 18585061

[138] LeVan P Tyvaert L Gotman J Modulation by EEG features of BOLD responses to interictal epileptiform discharges. Neuroimage (2010) 50:15–26 10.1016/j.neuroimage.2009.12.044 20026222 PMC3774651

[139] Grouiller F Thornton RC Groening K Spinelli L Duncan JS Schaller K With or without spikes: localization of focal epileptic activity by simultaneous electroencephalography and functional magnetic resonance imaging. Brain (2011) 134:2867–86 10.1093/brain/awr156 21752790 PMC3656675

[140] Elshoff L Groening K Grouiller F Wiegand G Wolff S Michel C The value of EEG-fMRI and EEG source analysis in the presurgical setup of children with refractory focal epilepsy. Epilepsia (2012) 53:1597–606 10.1111/j.1528-1167.2012.03587.x 22779700

[141] Morgan VL Li Y Abou-Khalil B Gore JC Development of 2dTCA for the detection of irregular, transient BOLD activity. Hum Brain Mapp (2008) 29:57–69 10.1002/hbm.20362 17290367 PMC2719759

[142] Hunyadi B Tousseyn S Mijovic B Dupont P Van Huffel S Van Paesschen W ICA extracts epileptic sources from fMRI in EEG-negative patients: a retrospective validation study. PLoS One (2013) 8:e78796 10.1371/journal.pone.0078796 24265717 PMC3827107

[143] Lopes R Lina JM Fahoum F Gotman J Detection of epileptic activity in fMRI without recording the EEG. Neuroimage (2012) 60:1867–79 10.1016/j.neuroimage.2011.12.083 22306797 PMC3753286

[144] Khatamian YB Fahoum F Gotman J Limits of 2D-TCA in detecting BOLD responses to epileptic activity. Epilepsy Res (2011) 94:177–88 10.1016/j.eplepsyres.2011.01.018 21353479 PMC3877045

[145] Kobayashi E Bagshaw AP Grova C Dubeau F Gotman J Negative BOLD responses to epileptic spikes. Hum Brain Mapp (2006) 27:488–97 10.1002/hbm.20193 16180210 PMC6871405

[146] Jacobs J Levan P Moeller F Boor R Stephani U Gotman J Hemodynamic changes preceding the interictal EEG spike in patients with focal epilepsy investigated using simultaneous EEG-fMRI. Neuroimage (2009) 45:1220–31 10.1016/j.neuroimage.2009.01.014 19349236

[147] Rathakrishnan R Moeller F Levan P Dubeau F Gotman J BOLD signal changes preceding negative responses in EEG-fMRI in patients with focal epilepsy. Epilepsia (2010) 51:1837–45 10.1111/j.1528-1167.2010.02643.x 20550554 PMC3771928

[148] Gotman J Pittau F Combining EEG and fMRI in the study of epileptic discharges. Epilepsia (2011) 52(Suppl 4):38–42 10.1111/j.1528-1167.2011.03151.x 21732941 PMC3753285

[149] Logothetis NK Pauls J Augath M Trinath T Oeltermann A Neurophysiological investigation of the basis of the fMRI signal. Nature (2001) 412:150–7 10.1038/35084005 11449264

[150] Pittau F Fahoum F Zelmann R Dubeau F Gotman J Negative BOLD response to interictal epileptic discharges in focal epilepsy. Brain Topogr (2013) 26:627–40 10.1007/s10548-013-0302-1 23793553 PMC4490905

[151] Voges N Blanchard S Wendling F David O Benali H Papadopoulo T Modeling of the neurovascular coupling in epileptic discharges. Brain Topogr (2012) 25:136–56 10.1007/s10548-011-0190-1 21706377

[152] Pollen DA Intracellular studies of cortical neurons during thalamic induced wave and spike. Electroencephalogr Clin Neurophysiol (1964) 17:398–404 10.1016/0013-4694(64)90163-4 14236822

[153] Fisher RS Prince DA Spike-wave rhythms in cat cortex induced by parenteral penicillin. I. Electroencephalographic features. Electroencephalogr Clin Neurophysiol (1977) 42:608–24 10.1016/0013-4694(77)90280-2 67022

[154] Masterton RA Harvey AS Archer JS Lillywhite LM Abbott DF Scheffer IE Focal epileptiform spikes do not show a canonical BOLD response in patients with benign rolandic epilepsy (BECTS). Neuroimage (2010) 51:252–60 10.1016/j.neuroimage.2010.01.109 20139011

[155] Grouiller F Vercueil L Krainik A Segebarth C Kahane P David O Characterization of the hemodynamic modes associated with interictal epileptic activity using a deformable model-based analysis of combined EEG and functional MRI recordings. Hum Brain Mapp (2010) 31:1157–73 10.1002/hbm.20925 20063350 PMC6871024

[156] Jacobs J Hawco C Kobayashi E Boor R Levan P Stephani U Variability of the hemodynamic response as a function of age and frequency of epileptic discharge in children with epilepsy. Neuroimage (2008) 40:601–14 10.1016/j.neuroimage.2007.11.056 18221891 PMC3793956

[157] Jacobs J Kobayashi E Boor R Muhle H Stephan W Hawco C Hemodynamic responses to interictal epileptiform discharges in children with symptomatic epilepsy. Epilepsia (2007) 48:2068–78 10.1111/j.1528-1167.2007.01192.x 17645544

[158] Storti SF Formaggio E Bertoldo A Manganotti P Fiaschi A Toffolo GM Modelling hemodynamic response function in epilepsy. Clin Neurophysiol (2013) 124:2108–18 10.1016/j.clinph.2013.05.024 23845895

[159] Hawco CS Bagshaw AP Lu Y Dubeau F Gotman J BOLD changes occur prior to epileptic spikes seen on scalp EEG. Neuroimage (2007) 35:1450–8 10.1016/j.neuroimage.2006.12.042 17399999

[160] Moeller F Siebner HR Wolff S Muhle H Granert O Jansen O Simultaneous EEG-fMRI in drug-naive children with newly diagnosed absence epilepsy. Epilepsia (2008) 49:1510–9 10.1111/j.1528-1167.2008.01626.x 18435752

[161] Pittau F Levan P Moeller F Gholipour T Haegelen C Zelmann R Changes preceding interictal epileptic EEG abnormalities: comparison between EEG/fMRI and intracerebral EEG. Epilepsia (2011) 52:1120–9 10.1111/j.1528-1167.2011.03072.x 21671923 PMC3771927

[162] Federico P Archer JS Abbott DF Jackson GD Cortical/subcortical BOLD changes associated with epileptic discharges: an EEG-fMRI study at 3 T. Neurology (2005) 64:1125–30 10.1212/01.WNL.0000156358.72670.AD 15824333

[163] Weinand ME Vascular steal model of human temporal lobe epileptogenicity: the relationship between electrocorticographic interhemispheric propagation time and cerebral blood flow. Med Hypotheses (2000) 54:717–20 10.1054/mehy.1999.0937 10859674

[164] Rouach N Koulakoff A Abudara V Willecke K Giaume C Astroglial metabolic networks sustain hippocampal synaptic transmission. Science (2008) 322:1551–5 10.1126/science.1164022 19056987

[165] Takano T Tian GF Peng W Lou N Libionka W Han X Astrocyte-mediated control of cerebral blood flow. Nat Neurosci (2006) 9:260–7 10.1038/nn1623 16388306

[166] Tian GF Azmi H Takano T Xu Q Peng W Lin J An astrocytic basis of epilepsy. Nat Med (2005) 11:973–81 10.1038/nm1277 16116433 PMC1850946

[167] Muthukumaraswamy SD Evans CJ Edden RA Wise RG Singh KD Individual variability in the shape and amplitude of the BOLD-HRF correlates with endogenous GABAergic inhibition. Hum Brain Mapp (2012) 33:455–65 10.1002/hbm.21223 21416560 PMC3374935

[168] Caballero-Gaudes C Van De Ville D Grouiller F Thornton R Lemieux L Seeck M Mapping interictal epileptic discharges using mutual information between concurrent EEG and fMRI. Neuroimage (2013) 68:248–62 10.1016/j.neuroimage.2012.12.011 23247187

[169] LeVan P Tyvaert L Moeller F Gotman J Independent component analysis reveals dynamic ictal BOLD responses in EEG-fMRI data from focal epilepsy patients. Neuroimage (2010) 49:366–78 10.1016/j.neuroimage.2009.07.064 19647798 PMC3779215

[170] Masterton RA Jackson GD Abbott DF Mapping brain activity using event-related independent components analysis (eICA): specific advantages for EEG-fMRI. Neuroimage (2013) 70:164–74 10.1016/j.neuroimage.2012.12.025 23266745

[171] Rodionov R De Martino F Laufs H Carmichael DW Formisano E Walker M Independent component analysis of interictal fMRI in focal epilepsy: comparison with general linear model-based EEG-correlated fMRI. Neuroimage (2007) 38:488–500 10.1016/j.neuroimage.2007.08.003 17889566

[172] Chaudhary UJ Duncan JS Lemieux L Mapping hemodynamic correlates of seizures using fMRI: A review. Hum Brain Mapp (2013) 34:447–66 10.1002/hbm.21448 22083945 PMC6870363

[173] Thornton RC Rodionov R Laufs H Vulliemoz S Vaudano A Carmichael D Imaging haemodynamic changes related to seizures: comparison of EEG-based general linear model, independent component analysis of fMRI and intracranial EEG. Neuroimage (2010) 53:196–205 10.1016/j.neuroimage.2010.05.064 20570736

[174] Tyvaert L Levan P Dubeau F Gotman J Noninvasive dynamic imaging of seizures in epileptic patients. Hum Brain Mapp (2009) 30:3993–4011 10.1002/hbm.20824 19507156 PMC3767605

[175] Sierra-Marcos A Maestro I Falcon C Donaire A Setoain J Aparicio J Ictal EEG-fMRI in localization of epileptogenic area in patients with refractory neocortical focal epilepsy. Epilepsia (2013) 54:1688–98 10.1111/epi.12329 23895643

[176] Chaudhary UJ Carmichael DW Rodionov R Thornton RC Bartlett P Vulliemoz S Mapping preictal and ictal haemodynamic networks using video-electroencephalography and functional imaging. Brain (2012) 135:3645–63 10.1093/brain/aws302 23250884

[177] Carmichael DW Vulliemoz S Rodionov R Thornton JS Mcevoy AW Lemieux L Simultaneous intracranial EEG-fMRI in humans: protocol considerations and data quality. Neuroimage (2012) 63:301–9 10.1016/j.neuroimage.2012.05.056 22652020

[178] Cunningham CB Goodyear BG Badawy R Zaamout F Pittman DJ Beers CA Intracranial EEG-fMRI analysis of focal epileptiform discharges in humans. Epilepsia (2012) 53:1636–48 10.1111/j.1528-1167.2012.03601.x 22881457

[179] Vulliemoz S Carmichael DW Rosenkranz K Diehl B Rodionov R Walker MC Simultaneous intracranial EEG and fMRI of interictal epileptic discharges in humans. Neuroimage (2011) 54:182–90 10.1016/j.neuroimage.2010.08.004 20708083

[180] Boucousis SM Beers CA Cunningham CJ Gaxiola-Valdez I Pittman DJ Goodyear BG Feasibility of an intracranial EEG-fMRI protocol at 3T: risk assessment and image quality. Neuroimage (2012) 63:1237–48 10.1016/j.neuroimage.2012.08.008 22902923

[181] Lenzi GL Jones T Frackowiak RS Positron emission tomography: state of the art in neurology. Prog Nucl Med (1981) 7:118–37 6976591

[182] Theodore WH Brooks R Sato S Patronas N Margolin R Di Chiro G The role of positron emission tomography in the evaluation of seizure disorders. Ann Neurol (1984) 15(Suppl):S176–9 10.1002/ana.410150734 6611120

[183] Garibotto V Heinzer S Vulliemoz S Guignard R Wissmeyer M Seeck M Clinical applications of hybrid PET/MRI in neuroimaging. Clin Nucl Med (2013) 38:e13–8 10.1097/RLU.0b013e3182638ea6 23242058

[184] Drzezga A Arnold S Minoshima S Noachtar S Szecsi J Winkler P 18F-FDG PET studies in patients with extratemporal and temporal epilepsy: evaluation of an observer-independent analysis. J Nucl Med (1999) 40:737–46 10319744

[185] Carne RP O’Brien TJ Kilpatrick CJ Macgregor LR Hicks RJ Murphy MA MRI-negative PET-positive temporal lobe epilepsy: a distinct surgically remediable syndrome. Brain (2004) 127:2276–85 10.1093/brain/awh257 15282217

[186] Nelissen N Van Paesschen W Baete K Van Laere K Palmini A Van Billoen H Correlations of interictal FDG-PET metabolism and ictal SPECT perfusion changes in human temporal lobe epilepsy with hippocampal sclerosis. Neuroimage (2006) 32:684–95 10.1016/j.neuroimage.2006.04.185 16762567

[187] Vinton AB Carne R Hicks RJ Desmond PM Kilpatrick C Kaye AH The extent of resection of FDG-PET hypometabolism relates to outcome of temporal lobectomy. Brain (2007) 130:548–60 10.1093/brain/awl232 16959818

[188] Magistretti PJ Cellular bases of functional brain imaging: insights from neuron-glia metabolic coupling. Brain Res (2000) 886:108–12 10.1016/S0006-8993(00)02945-0 11119692

[189] Kumar A Semah F Chugani HT Theodore WH Epilepsy diagnosis: positron emission tomography. Handb Clin Neurol (2012) 107:409–24 10.1016/B978-0-444-52898-8.00026-4 22938986

[190] Theodore WH Fishbein D Dubinsky R Patterns of cerebral glucose metabolism in patients with partial seizures. Neurology (1988) 38:1201–6 10.1212/WNL.38.8.1201 3135512

[191] Varrone A Asenbaum S Vander Borght T Booij J Nobili F Nagren K EANM procedure guidelines for PET brain imaging using [18F]FDG, version 2. Eur J Nucl Med Mol Imaging (2009) 36:2103–10 10.1007/s00259-009-1264-0 19838705

[192] Tepmongkol S Srikijvilaikul T Vasavid P Factors affecting bilateral temporal lobe hypometabolism on 18F-FDG PET brain scan in unilateral medial temporal lobe epilepsy. Epilepsy Behav (2013) 29:386–9 10.1016/j.yebeh.2013.08.017 24074882

[193] Chassoux F Rodrigo S Semah F Beuvon F Landre E Devaux B FDG-PET improves surgical outcome in negative MRI Taylor-type focal cortical dysplasias. Neurology (2010) 75:2168–75 10.1212/WNL.0b013e31820203a9 21172840

[194] Kudr M Krsek P Marusic P Tomasek M Trnka J Michalova K SISCOM and FDG-PET in patients with non-lesional extratemporal epilepsy: correlation with intracranial EEG, histology, and seizure outcome. Epileptic Disord (2013) 15:3–13 10.1684/epd.2013.0560 23531745

[195] Kagawa K Chugani DC Asano E Juhasz C Muzik O Shah A Epilepsy surgery outcome in children with tuberous sclerosis complex evaluated with alpha-[11C]methyl-l-tryptophan positron emission tomography (PET). J Child Neurol (2005) 20:429–38 10.1177/08830738050200050701 15971355

[196] Koepp MJ Richardson MP Brooks DJ Cunningham VJ Duncan JS Central benzodiazepine/gamma-aminobutyric acid A receptors in idiopathic generalized epilepsy: an [11C]flumazenil positron emission tomography study. Epilepsia (1997) 38:1089–97 10.1111/j.1528-1157.1997.tb01198.x 9579955

[197] Yankam Njiwa J Bouvard S Catenoix H Mauguiere F Ryvlin P Hammers A Periventricular [C]flumazenil binding for predicting postoperative outcome in individual patients with temporal lobe epilepsy and hippocampal sclerosis. Neuroimage Clin (2013) 3:242–8 10.1016/j.nicl.2013.07.008 24273709 PMC3814949

[198] Horsley V An address on the origin and seat of epileptic disturbance: delivered before the cardiff medical society. Br Med J (1892) 1:693–6 10.1136/bmj.1.1631.693 PMC2420022 20753617

[199] So EL O’Brien TJ Peri-ictal single-photon emission computed tomography: principles and applications in epilepsy evaluation. Handb Clin Neurol (2012) 107:425–36 10.1016/B978-0-444-52898-8.00027-6 22938987

[200] O’Brien TJ So EL Mullan BP Hauser MF Brinkmann BH Bohnen NI Subtraction ictal SPECT co-registered to MRI improves clinical usefulness of SPECT in localizing the surgical seizure focus. Neurology (1998) 50:445–54 10.1212/WNL.50.2.445 9484370

[201] Rowe CC Berkovic SF Austin MC Mckay WJ Bladin PF Patterns of postictal cerebral blood flow in temporal lobe epilepsy: qualitative and quantitative analysis. Neurology (1991) 41:1096–103 10.1212/WNL.41.7.1096 2067640

[202] Newton MR Berkovic SF Austin MC Rowe CC Mckay WJ Bladin PF Postictal switch in blood flow distribution and temporal lobe seizures. J Neurol Neurosurg Psychiatry (1992) 55:891–4 10.1136/jnnp.55.10.891 1431952 PMC1015183

[203] Dupont P Van Paesschen W Palmini A Ambayi R Van Loon J Goffin J Ictal perfusion patterns associated with single MRI-visible focal dysplastic lesions: implications for the noninvasive delineation of the epileptogenic zone. Epilepsia (2006) 47:1550–7 10.1111/j.1528-1167.2006.00628.x 16981872

[204] Lee SK Lee SY Yun CH Lee HY Lee JS Lee DS Ictal SPECT in neocortical epilepsies: clinical usefulness and factors affecting the pattern of hyperperfusion. Neuroradiology (2006) 48:678–84 10.1007/s00234-006-0106-z 16896909

[205] Patil S Biassoni L Borgwardt L Nuclear medicine in pediatric neurology and neurosurgery: epilepsy and brain tumors. Semin Nucl Med (2007) 37:357–81 10.1053/j.semnuclmed.2007.04.002 17707242

[206] Whiting P Gupta R Burch J Mota RE Wright K Marson A A systematic review of the effectiveness and cost-effectiveness of neuroimaging assessments used to visualise the seizure focus in people with refractory epilepsy being considered for surgery. Health Technol Assess (2006) 10:1.iii–250.iii 16487454 10.3310/hta10040

[207] Kim JT Bai SJ Choi KO Lee YJ Park HJ Kim DS Comparison of various imaging modalities in localization of epileptogenic lesion using epilepsy surgery outcome in pediatric patients. Seizure (2009) 18:504–10 10.1016/j.seizure.2009.04.012 19442538

[208] Schneider F Irene Wang Z Alexopoulos AV Almubarak S Kakisaka Y Jin K Magnetic source imaging and ictal SPECT in MRI-negative neocortical epilepsies: additional value and comparison with intracranial EEG. Epilepsia (2013) 54:359–69 10.1111/epi.12004 23106128

[209] Von Oertzen TJ Mormann F Urbach H Reichmann K Koenig R Clusmann H Prospective use of subtraction ictal SPECT coregistered to MRI (SISCOM) in presurgical evaluation of epilepsy. Epilepsia (2011) 52:2239–48 10.1111/j.1528-1167.2011.03219.x 22136078

[210] Son YJ Chung CK Lee SK Chang KH Lee DS Yi YN Comparison of localizing values of various diagnostic tests in non-lesional medial temporal lobe epilepsy. Seizure (1999) 8:465–70 10.1053/seiz.1999.0344 10627408

[211] Desai A Bekelis K Thadani VM Roberts DW Jobst BC Duhaime AC Interictal PET and ictal subtraction SPECT: sensitivity in the detection of seizure foci in patients with medically intractable epilepsy. Epilepsia (2013) 54:341–50 10.1111/j.1528-1167.2012.03686.x 23030361

[212] Zubler F Seeck M Landis T Henry F Lazeyras F Contralateral medial temporal lobe damage in right but not left temporal lobe epilepsy: a (1)H magnetic resonance spectroscopy study. J Neurol Neurosurg Psychiatry (2003) 74:1240–4 10.1136/jnnp.74.9.1240 12933926 PMC1738688

[213] Li LM Caramanos Z Cendes F Andermann F Antel SB Dubeau F Lateralization of temporal lobe epilepsy (TLE) and discrimination of TLE from extra-TLE using pattern analysis of magnetic resonance spectroscopic and volumetric data. Epilepsia (2000) 41:832–42 10.1111/j.1528-1157.2000.tb00250.x 10897154

[214] Goncalves Pereira PM Oliveira E Rosado P Relative localizing value of amygdalo-hippocampal MR biometry in temporal lobe epilepsy. Epilepsy Res (2006) 69:147–64 10.1016/j.eplepsyres.2006.01.012 16513328

[215] Simister RJ Mclean MA Barker GJ Duncan JS Proton MR spectroscopy of metabolite concentrations in temporal lobe epilepsy and effect of temporal lobe resection. Epilepsy Res (2009) 83:168–76 10.1016/j.eplepsyres.2008.11.006 19118980

[216] Pan JW Duckrow RB Gerrard J Ong C Hirsch LJ Resor SR Jr 7T MR spectroscopic imaging in the localization of surgical epilepsy. Epilepsia (2013) 54:1668–78 10.1111/epi.12322 23895497 PMC3938332

[217] Grova C Daunizeau J Kobayashi E Bagshaw AP Lina JM Dubeau F Concordance between distributed EEG source localization and simultaneous EEG-fMRI studies of epileptic spikes. Neuroimage (2008) 39:755–74 10.1016/j.neuroimage.2007.08.020 17945511 PMC3792086

[218] Boor R Jacobs J Hinzmann A Bauermann T Scherg M Boor S Combined spike-related functional MRI and multiple source analysis in the non-invasive spike localization of benign rolandic epilepsy. Clin Neurophysiol (2007) 118:901–9 10.1016/j.clinph.2006.11.272 17317297

[219] Formaggio E Storti SF Bertoldo A Manganotti P Fiaschi A Toffolo GM Integrating EEG and fMRI in epilepsy. Neuroimage (2011) 54:2719–31 10.1016/j.neuroimage.2010.11.038 21109007

[220] Van Houdt P Zijlmans M Different ways to analyze EEG-fMRI in focal epilepsy: does it matter? Clin Neurophysiol (2013) 124:2070–2 10.1016/j.clinph.2013.06.007 23849759

[221] Disbrow EA Slutsky DA Roberts TP Krubitzer LA Functional MRI at 1.5 tesla: a comparison of the blood oxygenation level-dependent signal and electrophysiology. Proc Natl Acad Sci U S A (2000) 97:9718–23 10.1073/pnas.170205497 10931954 PMC16931

[222] Hamandi K Powell HW Laufs H Symms MR Barker GJ Parker GJ Combined EEG-fMRI and tractography to visualise propagation of epileptic activity. J Neurol Neurosurg Psychiatry (2008) 79:594–7 10.1136/jnnp.2007.125401 18096681 PMC2571962

[223] Voets NL Beckmann CF Cole DM Hong S Bernasconi A Bernasconi N Structural substrates for resting network disruption in temporal lobe epilepsy. Brain (2012) 135:2350–7 10.1093/brain/aws137 22669081

[224] Kumar A Chugani HT The role of radionuclide imaging in epilepsy, part 2: epilepsy syndromes. J Nucl Med (2013) 54:1924–30 10.2967/jnumed.113.129593 24029652

[225] Kargiotis O Lascano AM Garibotto V Spinelli L Genetti M Wissmeyer M Localization of the epileptogenic tuber with electric source imaging in patients with tuberous sclerosis. Epilepsy Res (2014) 108:267–79 10.1016/j.eplepsyres.2013.11.003 24315017

[226] Kurian M Spinelli L Delavelle J Willi JP Velazquez M Chaves V Multimodality imaging for focus localization in pediatric pharmacoresistant epilepsy. Epileptic Disord (2007) 9:20–31 10.1684/epd.2007.0070 17307708

